# GDNF improves the cognitive ability of PD mice by promoting glycosylation and membrane distribution of DAT

**DOI:** 10.1038/s41598-024-68609-y

**Published:** 2024-08-01

**Authors:** Ma Chengcheng, An Panpan, Yan Yalong, Su Mingyu, Xu Wei, Chen Jing, Tang Chuanxi

**Affiliations:** 1 Xuzhou Key Laboratory of Neurobiology, Department of Neurobiology and Anatomy, Xuzhou Medical University, Xuzhou, 221004 Jiangsu China; 2 Jinhu County People’s Hospital, 160 Shenhua Avenue, Jinhu County, Huai’an City, Jiangsu China

**Keywords:** Glial cell line-derived neurotrophic factor, Parkinson's disease, Dopamine transporter, Glycosylation, GRASP65, Golgi, Cognitive neuroscience, Diseases of the nervous system, Neurotrophic factors, Synaptic transmission

## Abstract

The core of clinic treatment of Parkinson's disease (PD) is to enhance dopamine (DA) signaling within the brain. The regulation of dopamine transporter (DAT) is integral to this process. This study aims to explore the regulatory mechanism of glial cell line-derived neurotrophic factor (GDNF) on DAT, thereby gaining a profound understanding its potential value in treating PD. In this study, we investigated the effects of GDNF on both cellular and mouse models of PD, including the glycosylation and membrane transport of DAT detected by immunofluorescence and immunoblotting, DA signal measured by neurotransmitter fiber imaging technology, Golgi morphology observed by electron microscopic, as well as cognitive ability assessed by behavior tests. This study revealed that in animal trials, MPTP-induced Parkinson's Disease (PD) mice exhibited a marked decline in cognitive function. Utilizing ELISA and neurotransmitter fiber imaging techniques, we observed a decrease in dopamine levels and a significant reduction in the intensity of dopamine signal release in the Prefrontal Cortex (PFC) of PD mice induced by MPTP. Intriguingly, these alterations were reversed by Glial Cell Line-Derived Neurotrophic Factor (GDNF). In cellular experiments, following MPP + intervention, there was a decrease in Gly-DAT modification in both the cell membrane and cytoplasm, coupled with an increase in Nongly-DAT expression and aggregation of DAT within the cytoplasm. Conversely, GDNF augmented DAT glycosylation and facilitated its membrane transport in damaged dopaminergic neurons, concurrently reversing the effects of GRASP65 depletion and Golgi fragmentation, thereby reducing the accumulation of DAT in the Golgi apparatus. Furthermore, overexpression of GRASP65 enhanced DAT transport in PD cells and mice, while suppression of GRASP65 attenuated the efficacy of GDNF on DAT. Additionally, GDNF potentiated the reutilization of neurotransmitters by the PFC presynaptic membrane, boosting the effective release of dopamine following a single electrical stimulation, ultimately ameliorating the cognitive impairments in PD mice.Therefore, we propose that GDNF enhances the glycosylation and membrane trafficking of DAT by facilitating the re-aggregation of the Golgi apparatus, thereby amplifying the utilization of DA signals. This ultimately leads to the improvement of cognitive abilities in PD mouse models. Our study illuminates, from a novel angle, the beneficial role of GDNF in augmenting DA utilization and cognitive function in PD, providing fresh insights into its therapeutic potential.

## Introduction

Parkinson's disease (PD) is the second most common neurodegenerative disease in the world, occurring mainly in the elderly population. The primary pathological characteristic is degeneration of dopamine (DA) levels^1^. Most of DA neurons are clustered in two adjacent midbrain regions, the ventral tegmental area (VTA) and substantia nigra pars compacta (SNc)^2^. Therefore, the most obvious lesion in the brain of PD patients is the massive death of dopaminergic cells in the VTA and SNc. In addition to the well-known movement disorders, PD patients also have cognitive impairments, which aggravates the overall disability and seriously affects the daily life of patients^3^.

Dopaminergic neurons originating from the substantia nigra pars compacta (SNc) project to the striatum, forming the nigrostriatal pathway, which plays a critical modulatory role over the motor nuclei of the basal ganglia^4^. Dysfunction of this pathway is a hallmark feature of Parkinson’s Disease (PD), leading to motor symptoms such as bradykinesia, rigidity, and tremor^5^. Concurrently, the integrity of the nigrostriatal system is intricately linked to the functionality of the prefrontal cortex (PFC), a key region for executive function, working memory, and decision-making^6^. The PFC receives dense dopaminergic fiber projections from both the SNc and the ventral tegmental area (VTA), sustaining high-level cognitive performance^7^.

The disruption of dopaminergic modulation in the prefrontal cortex (PFC) is considered the primary culprit behind these cognitive impairments^8^, highlighting the significance of investigating dopaminergic transmission in the PFC. The prefrontal cortex (PFC) guides higher-order cognitive ability through representational knowledge, Dopamine receptors play a key role in PFC working memory and cognitive regulation^9^. Adequate levels of dopamine in the PFC are essential for normal cognitive ability and establish synaptic connections with dopaminergic neurons projected in the ventral tegmental area (VTA) of the midbrain^10^. Currently, the symptoms of PD are mainly alleviated by drug therapy, which works by enhancing the concentration of DA in the brain or by stimulating DA receptors associated with DA^11,12^.

DA transporter (DAT) is a transmembrane glycoprotein that is distributed on the surface of DA neurons and belongs to the Na^+^/Cl^-^ ion-dependent transporter gene family (SLC6A)^13^. Its main function is to regulate the intensity of DA signal and maintain DA homeostasis through DA reuptake^14^. The ability of DAT to take up DA depends on its expression level in the plasma membrane, and its membrane transport is correlated with glycosylation modification^15,16^. It has been found that in the striatum (Str) of PD rat models treated with 6-hydroxydopamine (6-OHDA) and rDAT-HEK cell models exposed to 1-methyl-4-phenylpyridinium iodide (MPP^+^), non-glycosylated DAT accumulates on the endoplasmic reticulum and Golgi, while glycosylated DAT decreases on both the endoplasmic reticulum and Golgi, as well as on the plasma membrane. It has been suggested that the lack of glycosylation modification of DAT may impede its transfer to the plasma membrane in DA neurons^16,17^. The aberrant function of DAT can lead to DA disorders, which may precipitate PD, schizophrenia, attention deficit hyperactivity disorder and other central nervous system pathologies^14,18–20^.

Golgi Reassembly Stacking Protein of 65 kDa (GRASP65) is a peripheral protein located on the Golgi apparatus, playing a crucial role in Golgi stack maintenance^17^. Under physiological conditions, GRASP65 tightly associates with the Golgi protein GM130, collectively preserving the structural and functional integrity of the cis-Golgi^21^. Studies have confirmed that, in addition to PD cellular and animal models, fragmented Golgi apparatuses can also be observed in the substantia nigra of PD patient brains, accompanied by reduced expression levels of GRASP65 protein^22^. These findings underscore the indispensable function of GRASP65 in sustaining Golgi apparatus integrity.

Glial cell-derived neurotrophic factor (GDNF) is an important trophic factor for maintaining the survival of DA neurons. It not only mitigates the neurodegeneration and motor deficits in the mouse model of PD but also ameliorates the cognitive deficits in aged rats^23–25^. The underlying mechanism of its action is not fully understood, although it is currently a potential drug for the treating PD. GDNF can increase total DAT expression in the striatum of a 6-OHDA-treated rat model of PD^26,27^. Our previous study found that decreased GDNF in the PFC triggered deficits in dopaminergic synaptic transmission, such as the degeneration of dopaminergic synaptic function, which may contribute to cognitive impairment in patients with PD^28^. However, the specific mechanism of GDNF on DAT in DA neurons still lacks evidence.

In our preceding research endeavors, our team observed alterations at the corticocortical termini of dopaminergic neurons^28^. This article shifts focus toward a more detailed investigation of neuronal terminal functionalities, specifically exploring aspects such as dopamine neurotransmitter uptake and release mechanisms. This study aimed to investigate the impact of GDNF on DAT expression and distribution, as well as its potential mechanism in a PD mouse model treated with 1-methyl-4-phenyl-1,2,3, 6-Tetrahydro-Pyridine (MPTP) and a PD cell model exposed to MPP + . In animal experiments, the Y-maze and Morris Water maze were used to assess the cognitive and learning abilities of mice^29–31^; and the Open field text was used to assess the locomotor abilities and exploratory behaviors of mice^32^. The findings provide a theoretical basis for exploring the mechanism of GDNF therapy for PD.

## Results

### GDNF reverses the defect of glycosylation and membrane distribution of DAT

**Figure 1:**
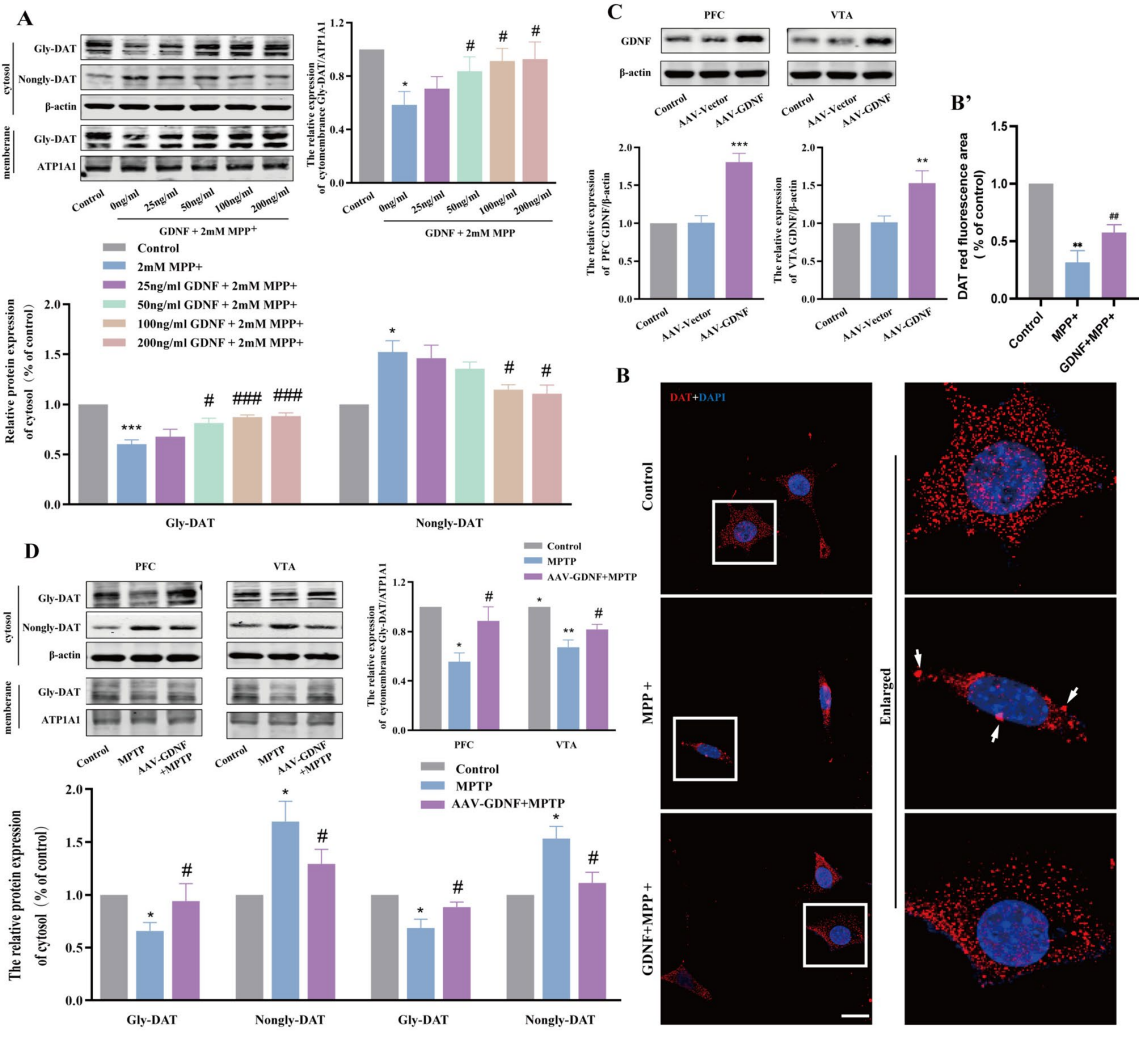
GDNF regulates DAT expression and distribution in damaged dopaminergic neurons of PD in vivo and in vitro. (**A**) Expression of gly-DAT and nongly-DAT in plasma membrane and cytosol of each group cell. (**B**) Immunofluorescence analysis of DAT in each group cell and enlarged view of a single cell. Scale bars equals 20 μm. (**C**) Expression of GDNF in PFC and VTA of each group mouse. (**D**) Expression of gly-DAT and nongly-DAT in PFC and VTA of each group mouse. (n = 3; *P < 0.05, **P < 0.01, ***P < 0.001 versus the control group; ^#^P < 0.05, ^##^P < 0.01, ^###^P < 0.001 versus the Mpp + or MPTP group).

To investigate the effect of GDNF on DAT in DA neurons, we evaluated the expression and localization of DAT in PD cells and mice using western blot and immunofluorescence techniques.In cell experiments, different concentrations of GDNF (0 ng/mL, 25 ng/mL, 50 ng/mL, 100 ng/mL, 200 ng/mL) and MPP were used Co-intervention of MES23.5 cells for 24 h, and then cell membrane cytoplasmic proteins were extracted to detect the expression of glycated DAT (Gly-DAT, 80 kDa) and non-glycated DAT (Nongly-DAT, 55 kDa). WB results showed that compared with Control group, the modification of cell membrane and cytoplasmic Gly-DAT decreased in MPP + group, while the expression of Nongly-DAT increased. GDNF increased the cell membrane and cytoplasmic Gly-DAT modification of PD, and decreased the cytoplasmic Nongly-DAT expression (Fig. 1A). Among them, the effect of 100 ng/mL GDNF has been obvious, and subsequent experiments use this concentration of GDNF and MPP + to intervene cells together.The distribution of DAT in cells could be observed by IF experiment, and the accumulation of DAT in cytoplasm could be observed in MPP + group compared with Control group. GDNF can reduce the accumulation of DAT in the cytoplasm of PD cells and redistribute DAT (Fig. 1B). The results of animal experiments are consistent with those of cell experiments. GDNF resulted in a decrease of Nongly-DAT in the cytoplasm of MPTP-induced mice with PD, while an increase of Gly-DAT expression in the cytoplasm and membrane of PFC and VTA (Fig. 1C,D).

### GDNF attenuates GRASP65 loss promotes Golgi aggregation and reduces DAT accumulation on Golgi

**Figure 2:**
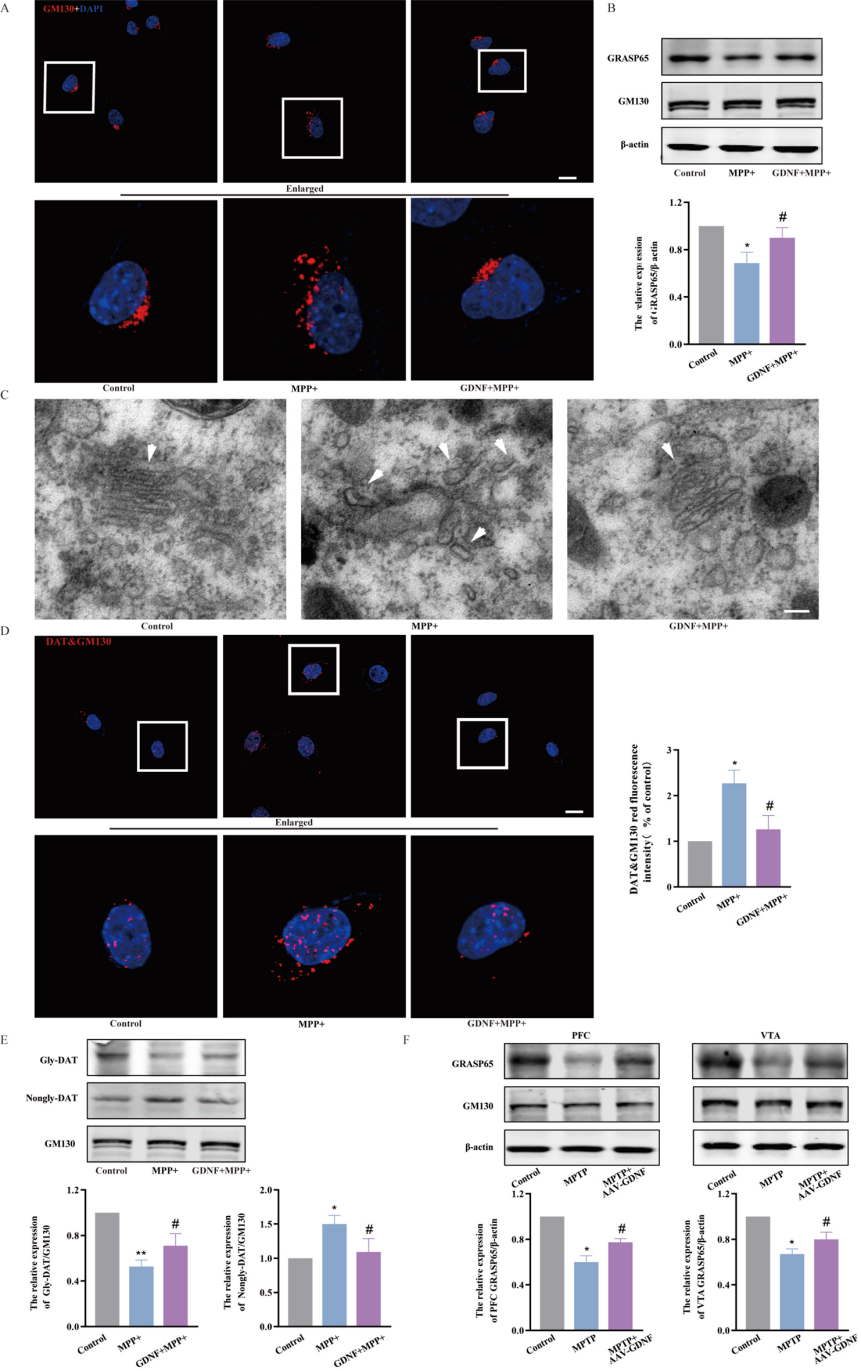
GDNF attenuates loss of GRASP65, promotes Golgi aggregation, and reduces DAT aggregation on the Golgi in dopaminergic-impaired neurons in vivo and in vitro. (**A**) The morphology of Golgi in each group by immunofluorescence; Scale bars equals 20 μm. (**B**) Expression of GRASP65 in each group cell. (**C**) The morphology of Golgi in each group by transmission electron microscopy; Scale bars equals 125 μm. (**D**) Proximity ligation assay of DAT and GM130 in each group cell; Scale bars equals 20 μm. (**E**) Expression of gly-DAT and nongly-DAT in Golgi protein of each group cell. (**F**) Expression of GRASP65 in PFC and VTA of each group mouse. (n = 3; *P < 0.05, **P < 0.01 versus the control group; ^#^P < 0.05, versus the Mpp + or MPTP group).

To further clarify elucidate the regulation of DAT membrane distribution by GDNF, we focused on the Golgi, which is associated with protein glycosylation and transport. The results showed that the expression of GRASP65 was decreased in MPP + -treated cells compared to the Control group, while there was no significant difference in GM130 expression. Treatment with GDNF prevented the loss of GRASP65 in PD cells, but did not significantly alter GM130 expression (Fig. 2B). The results from animal experiments were consistent with those from cell experiments, i.e., GDNF restored the loss of GRASP65 in PFC and VTA of PD mice (Fig. 2F). In addition, the Golgi of PD cells exhibited swelling, polycystization and fragmentation in the cytosol, which can be avoided by GDNF (Fig. 2A,C).

Subsequently, the glycosylation of DAT was observed to help clarify how GDNF regulates DAT membrane transport via the Golgi. Our results showed a decrease in Gly-DAT and an increase in Nongly-DAT in Golgi of PD cells, when compared to Control group. However, upon addition of GDNF, these changes were reversed (Fig. 2E). The results from proximity ligation assay revealed that, in comparison to the Control group, PD cells exhibited an augmented red fluorescence signal (representing accumulation of DAT in Golgi), The Negative control group without primary antibody showed no red light (Supplementary Fig. A), which was subsequently diminished by GDNF treatment (Fig. 2D).

### GRASP65 is crucial for the role of GDNF in DAT membrane distribution and Golgi remodeling

**Figure 3:**
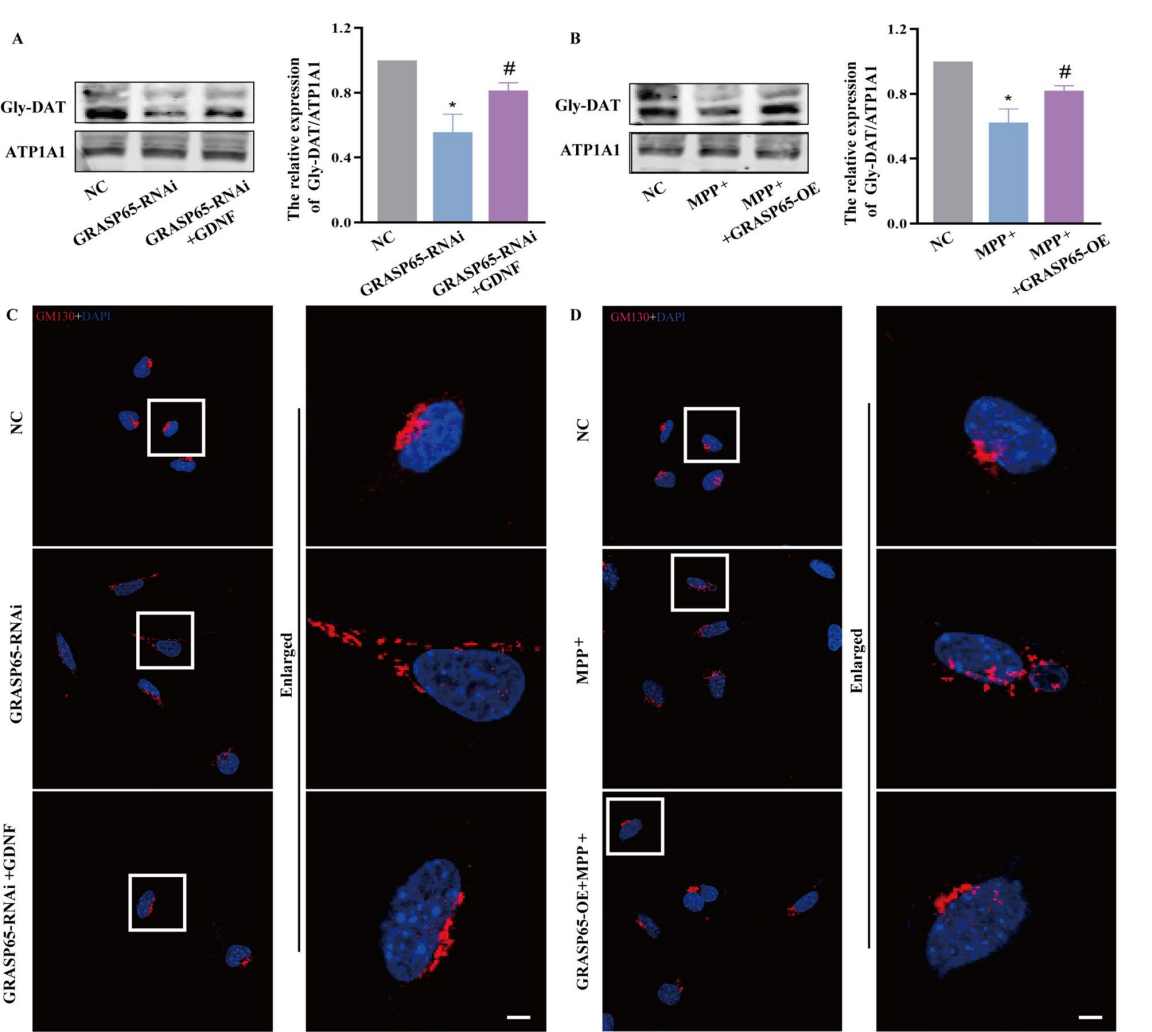
GRASP65 plays an important role in the regulation of DAT transport to membrane and Golgi remodeling in PD cells promoted by GDNF. (**A**) Expression of gly-DAT in each group cell. (**B**) The morphology of Golgi in each group by immunofluorescence; Scale bars equals 20 μm. (**C**,**D**) The immunofluorescence results of GM130 in each group, Scale bars equals 20 μm. (n = 3; *P < 0.05 versus the NC group; ^#^P < 0.05, versus the GRASP65i or Mpp + group).

We constructed MES23.5 cells stably knockdown and expressing GRASP65, to further validate the involvement of GRASP65 in GDNF-mediated regulation of DAT trafficking to the membrane in PD cells. The western blot analysis revealed that knockdown of GRASP65 not only reduced Gly-DAT protein expression in normal cells, but also impaired the up-regulation of Gly-DAT protein expression by GDNF in PD cells (Fig. 3A,B). Immunofluorescence results showed that knockdown of GRASP65 not only induced Golgi fragmentation in normal cells, but also abolished the Golgi remodeling mediated by GDNF in PD cells (Fig. 3C,D).

### GDNF overexpression in the PFC enhances DA signaling and ameliorates cognitive impairments in PD mice

Finally, based on the above data, we explore the impact of increased DAT membrane distribution by GDNF on DA signaling as well as cognitive deficits in PD. In this study, the cognitive abilities of the animal models were evaluated by open field test, Y-maze test and water maze test. GDNF-overexpressing virus (AAV-GDNF) was injected into the PFC of mice, and the negative control virus (AAV-Vector) was injected into the control group. Three weeks after injection, the virus infection fluorescence results showed that the virus infection was successful (Supplementary Fig. B).

**Figure 4:**
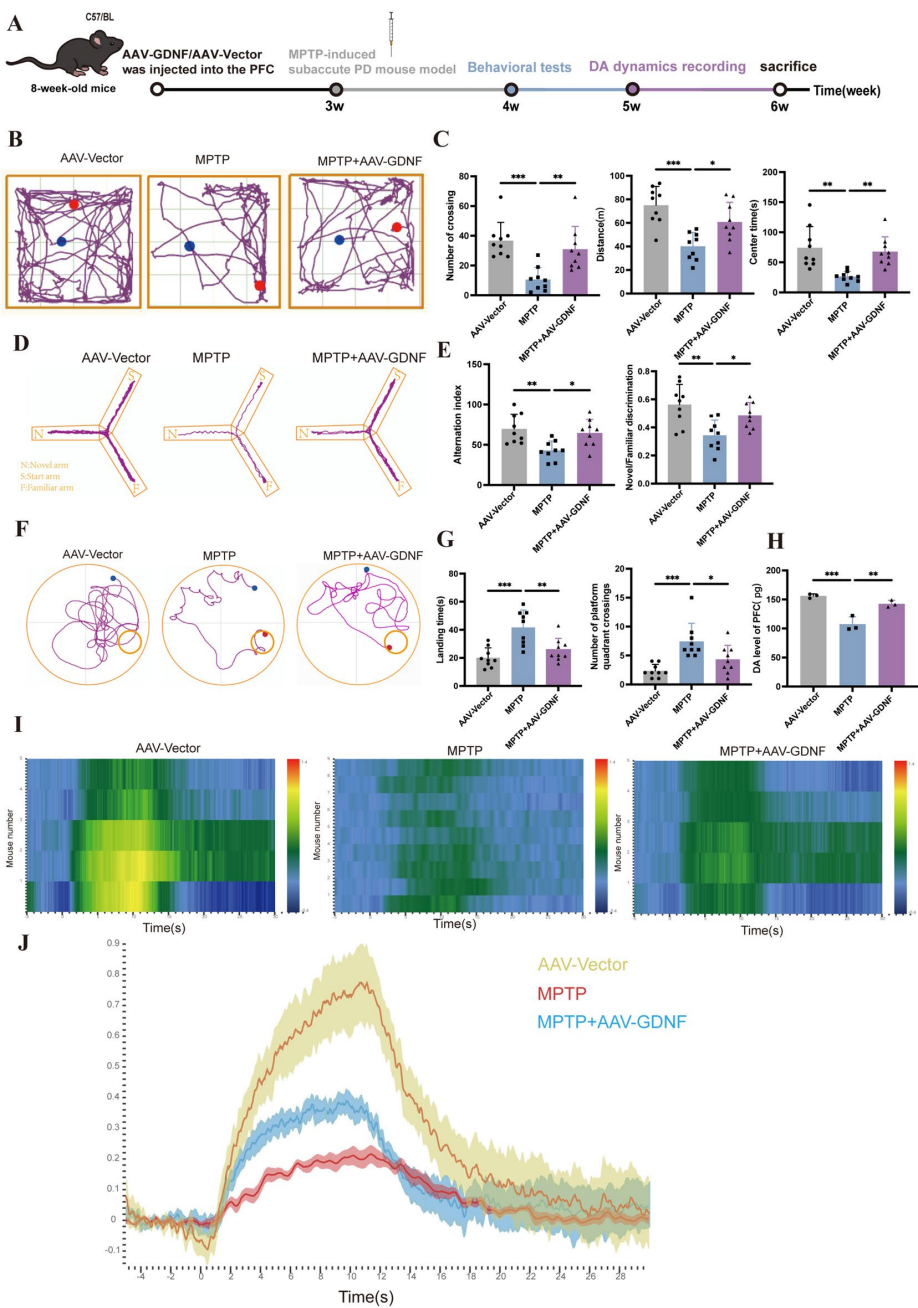
GDNF overexpression in the PFC enhances DA signaling and ameliorates cognitive impairments in PD mice. (**A**) Schematic diagram of mouse model establishment and behavioral experiment time axis in each group. (**B**) The representative movement track of each group of mice in open field experiment. (**C**) The results of the times of crossing the grid, the distance of movement and the center time in the open field were statistically plotted. (**D**) Representative movement trajectories of each group of mice in the Y maze. (**E**) The results of alternating index, Novel arm/Familiar arm discrimination were statistically analyzed. (**F**) Representative movement trajectories of each group of mice in the Water maze. (**G**) The results of landing time and number of platform quadrant crossings were statistically analyzed. (**B**–**G**), n = 9, *P < 0.05, **P < 0.01, ***P < 0.001) (**H**) The level of DA in PFC of mice in each group was detected by ELISA (n = 3, **P < 0.01, ***P < 0.001). (**I**) Heat map of DA signal release intensity change in PFC of each mouse detected by neurotransmitter fiber imaging technology; (**J**) Comparison of the intensity of DA signal release in PFC of mice in each group. (n = 3, ***P < 0.001).

Behavioral experiments were performed four weeks after injection of adenovirus overexpressing GDNF (Fig. 4A), and behavioral data were subjected to one-way ANOVA. The Open field test revealed that, compared to the AAV-Vector group, mice in the MPTP group exhibited significantly reduced total distance traveled and time spent in the center zone (Fig. 4B,C). Remarkably, in the MPTP + AAV-GDNF group, both parameters were notably increased, indicating impaired locomotor activity and decreased exploratory behavior in the MPTP group. The Y-maze test data showed a marked decrease in alternation index and novelty exploration ability in the MPTP group relative to the AAV-Vector group. However, the MPTP + AAV-GDNF group displayed a significant increase (Fig. 4D,E), suggesting a decline in cognitive function, exploratory drive towards novelty, and working memory in the MPTP group. Morris water maze text results (Fig. 4F,G) demonstrated that, compared to the MPTP group, the MPTP + AAV-GDNF group had a statistically significant difference in the time taken to reach the platform, with a notable reduction. Additionally, following GDNF intervention, PD mice crossed the target quadrant more frequently. This indicated that the cognitive ability and learning ability, spatial memory of MPTP group mice were significantly decreased. Following GDNF intervention, the aforementioned declines in cognitive capabilities, exploratory tendencies, and learning and memory functions were all ameliorated and effectively reversed.

After conducting a behavioral assessment to evaluate cognitive ability, we first measured DA levels in the PFC by ELISA. The results showed a significant decrease in DA levels in the PFC of MPTP group compared to AAV-Vector group. DA levels in the PFC were significantly elevated in the MPTP + AAV-GDNF group compared to the MPTP group (Fig. 4H). The following experiments were performed to investigate alternations in DA signaling in the PFC using fiber imaging techniques (Fig. 4I,J). The results showed that the continuous stimulation of VTA electrode (10S), resulted in a sustained enhancement of DA signal in the mouse brain across all groups. However, the DA signal in the PFC was significantly attenuated in the MPTP group compared to AAV-Vector group. DA signal in the PFC was significantly augmented in the MPTP + AAV-GDNF group compared to the MPTP group. In addition, we examined TH expression in the substantia nigra by WB (Supplementary Fig. C) and IF (Supplementary Fig. D). TH expression in the substantia nigra of MPTP mice group was significantly lower than that of AAV-Vector group mice and was significantly restored after GDNF intervention.

## Discussion

Parkinson’s disease (PD) is a prevalent chronic neurodegenerative disorder characterized by the loss of dopaminergic neurons in the substantia nigra^33^. The resulting motor impairments and cognitive deficits significantly interfere with patients’ daily lives and add considerable burden to families^34,35^. Consequently, there is an urgent need for therapeutic approaches capable of slowing or halting the progression of PD.Prior investigations have centered on the role of Glial cell-derived neurotrophic factor (GDNF) within the mesocorticolimbic dopaminergic system, encompassing regions such as the prefrontal cortex (PFC), ventral tegmental area (VTA), and nucleus accumbens; however, these studies have yet to elucidate the precise impact of GDNF in the PFC on cognitive function in PD patients and the underlying mechanisms. Preliminary data from our research group’s animal experiments confirm that in the subacute MPTP mouse model of PD, GDNF levels are diminished in the PFC alongside a notable reduction in dopamine concentration^28^, a change that GDNF administration is capable of reversing.

Based on previous studies, the present study delves into the role of GDNF in DAT glycosylation and membrane distribution, as well as its ultimate impact on DA signal as well as cognitive ability in PD. Our results suggest that GDNF ameliorated the defect of DAT glycosylation modification to increase its membrane distribution by enhancing Golgi aggregation, followed by the enhancement of DA signaling, and ultimately ameliorating cognitive impairment in PD.

The MPTP-induced PD symptoms and their impacts on brain structures closely mirror those observed in human PD patients, rendering MPTP-induced animal models and MPP + -induced cellular models indispensable tools for unraveling the pathogenic mechanisms and potential therapies for PD. Notably, in MPTP models, abnormal accumulation of α-synuclein has been substantiated, and α-synuclein has been shown to regulate the trafficking of the DAT^36^. With these considerations in mind, this study employs an MPTP-induced mouse model and MPP + -treated MES23.5 cell lines to construct PD models. The primary objective is to comprehensively investigate how GDNF enhances cognitive function in PD mice by facilitating DAT glycosylation and membrane distribution, along with elucidating its precise mechanisms of action within the mesocortical dopaminergic neurons.

Numerous studies have demonstrated the association between DAT and a variety of neurological and psychiatric diseases, and its aberrant functioning leads to DA disorders, which subsequently induce a variety of central nervous system diseases including PD^14,37^. Middle-aged GDNF heterozygous mice have enhanced DAT activity and DA reuptake^38^, and injection of GDNF factor or plasmid into the striatum attenuated the loss of DAT and DA in the striatum of PD rat models treated with 6-OHDA^27^. However, administration of viruses overexpressing GDNF in the rat striatum resulted in a decrease in DAT activity and DA reuptake, with no effect on the expression of tyrosine hydroxylase (TH) in the striatal pathway^39^. The inconsistency in these findings regarding the regulation of DAT and DA by GDNF may be attributed to differences in the study model and the age of model. Our results suggest that GDNF improves defective DAT glycosylation and increases the membrane distribution of DAT in PD cells, as well as yielding consistent results in the prefrontal cortex (PFC) and the ventral tegmental area (VTA) of PD mice. DA neurons projecting from VTA to PFC release an adequate amount of DA to ensure proper execution of cognitive abilities^9,10,40^. Previous studies from our group have shown that increasing GDNF in the mPFC significantly enhanced dendritic branching and the length and density of dendritic spines in Parkinson disease mice, thereby restoring the morphological structure and synaptic plasticity of pyramidal neurons. These findings suggest that GDNF intervention may promote synaptic plasticity in the mPFC, contributing to the development of excitatory microcircuits^41^. Our results also confirm that GDNF enhances DA signaling in the PFC and ameliorates cognitive impairment in PD mice.

The potential mechanism underlying the regulation of DAT translocation to the membrane by GDNF may be attributed to alterations in Golgi. Golgi is an important site for intracellular protein synthesis, processing and modification, and its fragmentation is a common feature of most neurodegenerative diseases^42–44^. Golgi reassembly stacking protein of 65 kDa (GRASP65) is a crucial component involved in the process Golgi stacking, playing pivotal roles in maintaining Golgi integrity, facilitating protein glycosylation modification, regulating mitosis and controlling apoptosis^45–48^. Knockdown of GRASP65 in Hela cells results in disassembly of the cis-Golgi stack, fragmented Golgi distribution, significant reduction in glycoproteoglycans, and impaired glycosylation, all of which can be restored by addition of exogenous GRASP65^46,49^. In substantia nigra (SN) of PD patients, reduced GRASP65 protein expression and fragmented Golgi were observed^22^. Moreover, our previous findings have shown that knockdown of Golgin160 or GM210 leads to fragmented Golgi in U251 cells, whereas addition of GDNF can promote Golgi aggregation^50^. Based on prior research, disruption of the Golgi apparatus reduces the transport of the dopamine transporter (DAT) to the membrane^51^, leading us to hypothesize that a decrease in GRASP65 would similarly impede the trafficking of DAT to the plasma membrane. Interestingly, Based on the above evidence, we hypothesize that the decrease of GRASP65 in DA neurons of PD may lead to the fragmentation and abnormal function of Golgi, which in turn affects the defective glycosylation modification of DAT and its transport to the membrane. In our study, a reduction in GRASP65 was observed in both PFC and VTA of PD mice as well as PD cells, which can be reversed by GDNF; moreover, GDNF alleviated Golgi fragmentation and reduced the accumulation of non-glycosylated DAT in the Golgi on PD cells. Meanwhile, the results of GM130 showed no significant changes, which is consistent with the unchanged GM130 expression in the SN of PD patients^22^. By further constructing DA cells stably knockdown and expressing GRASP65, we demonstrated that GDNF promotes Golgi aggregation and enhanced DAT glycosylation to facilitate DAT transport to the plasma membrane by upregulating GRASP65 in PD cells.

We added electrophysiological experiments in the CA1 region of mouse hippocampus and recorded field excitatory postsynaptic potentials (fEPSPs) in the CA1 region of hippocampus of mice in the MPTP group and MPTP + GDNF group. Statistical analysis showed a significant difference between the two (Supplementary Fig. E, the experimental method is in the supplementary experiment), suggesting that neurons in the hippocampal region were also protected after GDNF injection in prefrontal functional areas. This is fully consistent with the retrograde protective properties of GDNF expressed in our discussion that GDNF injection in prefrontal functional areas promotes the survival of midbrain dopaminergic neurons. Since dopaminergic neurons from the VTA also project to the hippocampal CA1 region, previous studies by our group have shown that increasing the survival of midbrain VTA dopaminergic neurons (Such as through sports) leads to enhanced hippocampal dopaminergic projections^52^. Thus, our current study and the hippocampal electrophysiologic results in the accompanying figures support this idea.

**Figure 5:**
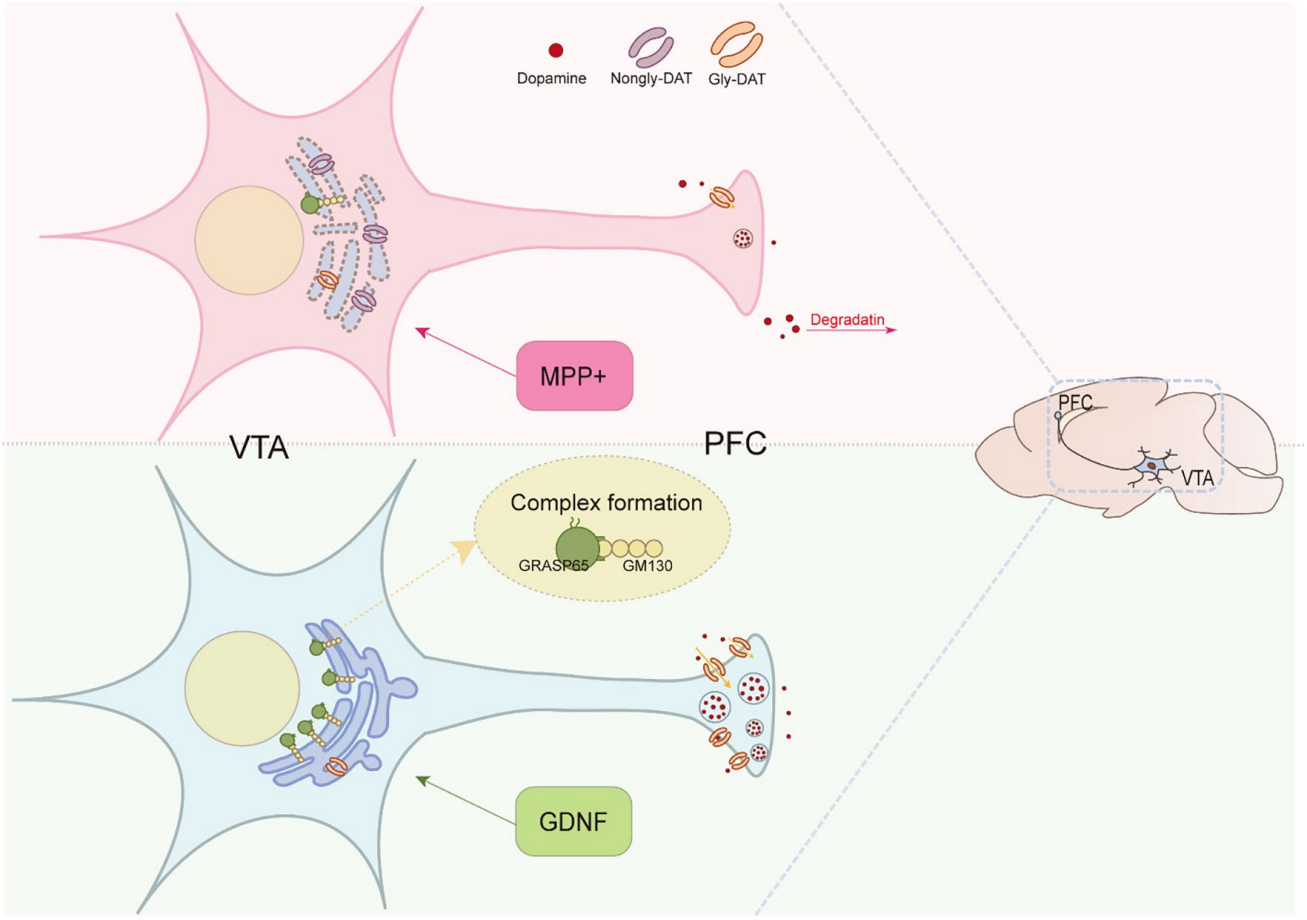
Graphical diagram depicting GDNF improves cognitive function in PD mice by promoting glycosylation and membrane distribution of DAT.

In conclusion, we propose that GDNF improves the cognitive ability of PD mice by promoting glycosylation and membrane distribution of DAT, which is achieved by upregulating GRASPER65 to promote Golgi aggregation (Fig. 5). In this study, we used DAT glycosylation as a breakthrough point to explore the intrinsic mechanism of GDNF to promote DAT membrane transport in DA neurons, which not only deepens our understanding of the potential of GDNF for Parkinson's disease, but also has implications in the field of clinical application. DAT SPECT imaging using FP-CIT provides a non-invasive means of monitoring the absence of nigrostriatal dopaminergic neuronal endings, a hallmark of Parkinson's disease^53^. In addition, the unique binding pattern of DAT can be used as a diagnostic tool for a variety of Parkinsonian syndromes, including Parkinson's disease dementia (PDD)^54^. Our findings suggest that interventions to optimize DAT function through GDNF administration have the potential to translate into clinical applications. By assessing treatment efficacy through DAT SPECT imaging, clinicians may gain a valuable biomarker for monitoring disease progression and treatment response. These approaches could revolutionize the management of Parkinson's disease by customizing treatments based on an individual's DAT profile and cognitive health.

## Materials and methods

### MPTP-induced subacute PD mouse model

Male, 4-week-old, C57BL/6 mice (22–25 g), were purchased from Shandong Jinan Pengyue Laboratory Animal Breeding Co., LTD (Jinan, China SCXY(鲁)2019 0003). Mice were housed at the Laboratory Animal Center of Xuzhou Medical University. The ambient temperature was controlled at about 25 °C, and the animals were fed standard diet and purified water for 12 h of day and night cycle. All the mice were randomly divided into three groups (n = 9/group): vector group (Vector), MPTP-treated group (MPTP) and adenoviral injection of GDNF overexpressing MPTP-treated group (MPTP + GDNF). MPTP group mice were intraperitoneally injected with MPTP (30 mg/kg, M0896, Sigma St. Louis, MO, USA) solution at 11:00 am once a day for 5 consecutive days. The mice in the Control group were given the same volume of saline. Four weeks after adenoviral injection of GDNF overexpression, behavioral analysis was performed. After anaesthetizing mice with Alfaxan®, fiber optic imaging experiments were performed. After the mice were euthanized, ELISA, western blotting and immunofluorescence assays were performed. All animal experiments were approved by the Animal Experiment Committee of Xuzhou Medical University (approval Nos. L20210226140 [March 2, 2021] and L20210226312 [February 28, 2021]). All animal experiments were conducted in strict accordance with the "Guiding Opinions on the Treatment of Laboratory Animals" promulgated by the Ministry of Science and Technology of China. All methods were performed in accordance with relevant guidelines and regulations. Animal studies were reported in accordance with ARRIVE guidelines.

### MPP^+^ /GDNF-induced PD cell model

MES23.5 was bought from Shenzhen Haodi Huatuo Biotechnology Co., LTD (Shenzhen, China). Cells were cultured in petri dishes in DMEM medium containing 10% FBS and 1% penicillin–streptomycin in an atmosphere of 5% CO_2_ at 37 °C. The MES23.5 treated with different concentrations of GDNF (0 ng/mL, 25 ng/mL, 50 ng/mL, 100 ng/mL, 200 ng/mL, G1401, Sigma St. Louis, MO, USA) and MPP^+^ (2 mM, D048, Sigma St. Louis, MO, USA) for 24 h were selected for western blot analysis. The MES23.5 treated with GDNF (100 ng/mL) and MPP^+^ (2 mM) for 24 h were selected for western blot analysis, immunofluorescence, proximity ligation assay, and transmission electron microscope.

### Cell experiment

MES23.5 cells were transfected with purified virus (LV-Gorasp1-RNAi, from Gene Shanghai, China), and then selected by medium containing puromycin. The specific groups were NC group, GRASP65-RNAi GRASP65-RNAi + GDNF group. Stable GRASP65 knockdown cells were treated with nothing or GDNF (100 ng/ml) for 24 h, and then samples were collected for western blot analysis and immunofluorescence. All cell experiments are repeated three or more times.

### Stereotactic injection into mouse brain

Mice were divided into three groups : (1) AAV-Vector group, mice were injected with AAV-Vector in the left brain and saline intraperitoneally; (2) MPTP group, mice were injected with AAV-Vector in the left brain and MPTP intraperitoneally; (3) MPTP + AAV-GDNF group, mice were injected with AAV-GDNF in the left brain, and MPTP intraperitoneally. AAV-GDNF (2ul, from Gene Shanghai, China) was injected into the left PFC of mice with the coordinates 1.78 mm anterior to bregma, 0.25 mm lateral to the midline and 2.25 mm ventral to the skull surface. The negative control group was injected with AAV-vector at the same position and the same dose. After 3 weeks, mice were intraperitoneally injected with MPTP (30 mg/kg) for 5 consecutive days, and mice in the AAV-Vector group were injected with the same volume of 0.9% saline. Seven days later, the mice were sacrificed, and the brain tissue was taken for western blot analysis.

### Behavioral testing

#### Open field text

The open field test was performed on each group of mice, and the movement distance and penetration ability of mice in the open field were counted.

Voluntary movement, exploration of new environment, tension, mania, anxiety and depression were observed by the number and time of staying in the central area^32^. The experimental data were recorded by ANY-maze software small animal behavior trajectory detection and analysis system.

#### Y maze text

The Y maze is used to test discriminative learning, working memory and reference memory in animals^29^. During the training period, the new arm was blocked by the baffle, and the mice were placed by the starting arm and moved freely in the starting arm and other arms for 10 min. One hour later, the detection period was entered, the new alien arm partition was pulled out, and the mice were placed in the starting arm and moved freely in the three arms for 5 min. The time and shuttle times of each mouse in each arm within 5 min were recorded by video recording.

#### Morris water maze text

Morris Water Maze can detect animal cognitive ability^30^. The learning ability of mice was assessed. In the ANY-Maze software, the water maze is divided into five quadrants, of which the fifth is in the fourth quadrant. It is a detachable circular platform.

The first day of the induction period: inject clear tap water into the device, ensure that the height of the platform is greater than the horizontal level, and put the mice into the first quadrant to the fourth quadrant successively. If the mice find the platform within 1 min, and stay on the platform for 1 min, then guide the mice to find the platform and stay on the platform for 15 s.

Day 2 to 6 training period: Add clear tap water to the device until it is 1 cm above the platform. Then add milk powder to make the water cloudy, cannot see the platform. Then, as above, put mice in turn.

Wait for 7 days test period: the operation is the same as above. Finally, the computer records the movement track, observes the fatigue and records the time the mice stay in the second quadrant and the time they first arrive in the fifth quadrant.

### Western blot

For total protein extraction, cells and mouse brain tissues were homogenized in RIPA lysis buffer. The lysate was incubated on ice for 30 min and then centrifuged at 14,000 rpm at 4 °C for 30 min. Cell membrane proteins and cytoplasmic proteins were extracted from cells and mouse brain tissues according to the manufacturer's protocol of the cell membrane proteins and cytoplasmic proteins Extraction kit. Proteins on the Golgi apparatus was extracted according to the manufacturer's protocol of Golgi protein kit. Protein concentration was detected by BCA protein concentration kit. Proteins in the samples were separated by SDS-PAGE and then transferred to NC membranes. The membranes were blocked with 5% skim milk for 1 h, and then the following primary antibodies were incubated overnight at 4 °C: Anti-β-actin (1:5000, 60008-1-1 g, Proteintech, Wuhan, China) , Anti-ATP1A1 (1:20,000, 14418-1-AP, Proteintech, Wuhan, China), Anti-GDNF (1:1000, SAB1401150, Sigma St. Louis, MO, USA), Anti-DAT (1:1000, MAB369, for glycosylated DAT labelling; sc-32259, for non-glycosylated DAT labelling, Santa, CA, USA), Anti-GRASP65 (1:1000, 10747-2-AP, Proteintech, Wuhan, China), Anti-GM130 (1:1000, NBP2-53,420, Novus , Colorado, USA). Finally, the target proteins were detected after fluorescent secondary antibodies (1:10,000) incubated for 2 h in the dark. All blots were processed in parallel and derived from the same experiment. The specific antibody dilution ratio and other information in this experiment can be seen in the supplementary figure (Experimental supplement, Table 1).

### Immunofluorescence

Cells in each group were fixed with 4% formaldehyde for 20 min, and then treated with 0.3% TritonX-100 for 20 min. After that, the cells were blocked with immunostaining blocking solution for 20 min and then incubated with Anti-DAT (1:250) and Anti-GM130 (1:300) overnight at 4 °C. The next day, the cells were incubated with fluorescent secondary antibodies (1:500) for 2 h and then stained with DAPI for 5 min in the dark. Finally, the images were obtained with a confocal microscope. The specific antibody dilution ratio and other information in this experiment can be seen in the supplementary figure (Experimental supplement, Table 2).

### Proximity ligation assay

The Duolink™ PLA kit (DUO92101) from Sigma (St. Louis, MO, USA). Cells in each group were fixed with 4% formaldehyde for 20 min, and then treated with 0.3% TritonX-100 for 20 min. After that, the cells were blocked with immunostaining blocking solution for 20 min and then incubated with Anti-DAT(1:250) and Anti-GM130(1:300) overnight at 4 °C. The next day, the samples were incubated with probe PLUS (1:5) and MINUS (1:5) for 1 h, and then treated with ligase (1:40) for 30 min and polymerase (1:80) for 100 min at 37 °C. Finally, the samples were stained with DAPI for 5 min and then obtained with a confocal microscope.

### Transmission electron microscope

Cells in each group were fixed with 2.5% glutaraldehyde overnight. The next day, the samples were fixed with 1% osmic acid for 2 h. After progressive dehydration, the samples were infiltrated with different concentrations of resin (33%, 66%, 100%) for 2 h every step. Finally, after embedding and staining, the samples were observed by transmission electron microscopy.

### Enzyme-linked immunosorbent assay

Detection of mouse brain tissue supernatant by enzyme-linked immunosorbent assay(DA ELISA Kit [mice]: Cat#MM-0626M1, MEIMIAN, Yancheng, Jiangsu, China). The protocol was performed according to manufacturer instructions. The optical density values were detected using a microplate reader (BioTek, Hercules, CA, USA).

### DA dynamics recording–neurotransmitter fiber optic imaging

Drill through the skull case with a skull drill and fix the microinjector that inhales AAV-GDNF (2uL) & AAV-CamkiA-DA1H (500nL) virus solution on the microinjection pump. The needle was slowly injected into the borehole 2.25 mm. The control group was AAV-Vector (2uL) & AAV-Camkia-DA1H (500 nL) virus solution. Keep the needle for 5 min and adjust the micro-injection pump to inject the virus solution at a speed of 400nL/min. After the injection, keep the needle for 8 min, and then slowly remove the syringe. Insert the end of the optical fiber slowly to a depth of 2.05 mm in the same position, and fix the optical fiber with dental cement; The fontanel is 3.20 mm after the zero point, 0.45 mm off the left side, marked; Use a skull drill to break the skull case at the mark, slowly insert the end of the electrode to a depth of 4.30 mm, and fix the electrode with dental cement; The mice were moved into the incubator, and when they were conscious and able to move freely, the mice were put back into the cage for feeding, and the activities of the mice were closely observed. After the molding was completed, the changes of fluorescence signals before and after electrical stimulation VTA (100 Hz, wave width 1 ms, duration 10 s, intensity 100uA) were recorded by optical fiber recording system (Qianaoxing Kenanjing Biotechnology Co., LTD.).

### Statistical analysis

The experimental data were analyzed using SPSS 22.0, and the quantitative data were presented as mean ± standard errors. The data were assessed for the homogeneity of variance using Levene's test, as well as for normality using Shapiro–Wilk tests. If the data met the assumptions of the parametric test, comparisons between two groups utilized Student's t-test, whereas comparisons among multiple groups employed one-way analysis of variance (ANOVA) followed by Tukey's post-hoc test. The criterion for determining a statistically significant difference was set at P < 0.05. The statistical graphs were generated using GraphPad 8.0.2 and Adobe Illustrator 23.0.3.

### Supplementary Information

Supplementary Information.

Supplementary Figures.

Supplementary Figure 1.

## Data Availability

All data generated during this study are included in this published article (and its Supplementary information files).
