# Supplementary material for: GDNF improves the cognitive ability of PD mice by promoting glycosylation and membrane distribution of DAT
Source: Sci Rep. 2024 Aug 1;14:17845. doi: 10.1038/s41598-024-68609-y (PMC11294596; doi:10.1038/s41598-024-68609-y)
Supplement: Supplementary file 1 — Supplementary Information. [file 41598_2024_68609_MOESM1_ESM.pdf]

GDNF improves the cognitive ability of PD mice by promoting glycosylation and membrane distribution of DAT

Ma Chengcheng[a*], An Panpan[a*], Yan Yalong[a], Su Mingyu[a], Xu Wei[b], Chen Jing[a#], Tang Chuanxi[a#]

Experimental supplement

Table1

Western Blot	Name of antibody	Manufacturer	Batch number	Working dilutions
**Primary antibodies**	Anti-β-actin	Proteintech	60008-1-1g	1:20000
	Anti-ATP1A1	Proteintech	14418-1-AP	1:20000
	Anti-GDNF	Sigma	SAB1401150	1:1000
	Anti-DAT	Santa	sc-32259	1:1000
	Anti-DAT	MERCK	MAB369	
	Anti-GRASP65	Proteintech	10747-2-AP	1:1000
	Anti-GM130	Novus	NBP2-53420	1:1000
	Anti-TH	Santa	sc-374048	1:1000
**Secondary antibodies**	IRDye® 680RD Goat anti-Mouse IgG	LI-COR	926-68070	1:20000
	IRDye® 800CW Goat anti-Rabbit IgG	LI-COR	926-32211	1:20000

Table2

Immunofluorescence	Name of antibody	Manufacturer	Batch number	Working dilutions
**Primary antibodies**	Anti-DAT	Santa	MAB369 ; sc-32259	1:250
	Anti-GM130	Novus	NBP2-53420	1:300
	Anti-TH	Abcam	ab112	1:750
**Secondary antibodies**	goat anti-mouse Alexa Fluor® 594	Abcam	ab150116	1:500
	goat anti-rabbit Alexa Fluor® 488	Abcam	ab150077	1:500

Electrophysiological whole-cell patch-clamp recordings:

After anaesthetizing mice with Alfaxan®, and transverse hippocampus slices (400 μm thickness) were prepared using a vibratome in ice-cold ACSF (Sigma-Aldrich, St. Louis, Missouri, USA). Slices were placed in the recording chamber (RWD Life Science Co., Ltd., Shenzhen,China), which was superfused (3 – 4 mL/min) with ACSF at 32 – 34 ° C. The location of the pyramidal cell layer in the CA1 region was determined using microscopy, and the brain slices were fixed with anchors.The borosilicon patch clamp electrode is filled with the electrode liquid and the electrode resistance is 3~5 MΩ.Record the hippocampus CA1 area field excitatory postsynaptic potential (fEPSP). The test stimulus consisted of a single phase constant current pulse of 0.1 ms (adjusted intensity to produce 50% of the maximum response) at a frequency of 0.033Hz. Double pulse stimulation was performed at intervals of 20, 40, 60, 80, 100, 150, 200, 400, 600ms. Calculate the ratio of the second and first fEPSP fluctuations. The stimulus intensity was determined by measuring the 50% maximum rising fEPSP phase slope. Stabilize the recorded signal for 15 minutes as a baseline. All dissolve the drug in the ACSF and administer it by switching the infusion from the control ACSF to the drug-containing ACSF. High frequency stimulation (HFS; 100 Hz, 1000 ms x 2, interval 30 s). LTP was recorded 30 minutes after HFS.
