# Supplementary material for: GDNF improves the cognitive ability of PD mice by promoting glycosylation and membrane distribution of DAT
Source: Sci Rep. 2024 Aug 1;14:17845. doi: 10.1038/s41598-024-68609-y (PMC11294596; doi:10.1038/s41598-024-68609-y)

fig1: B

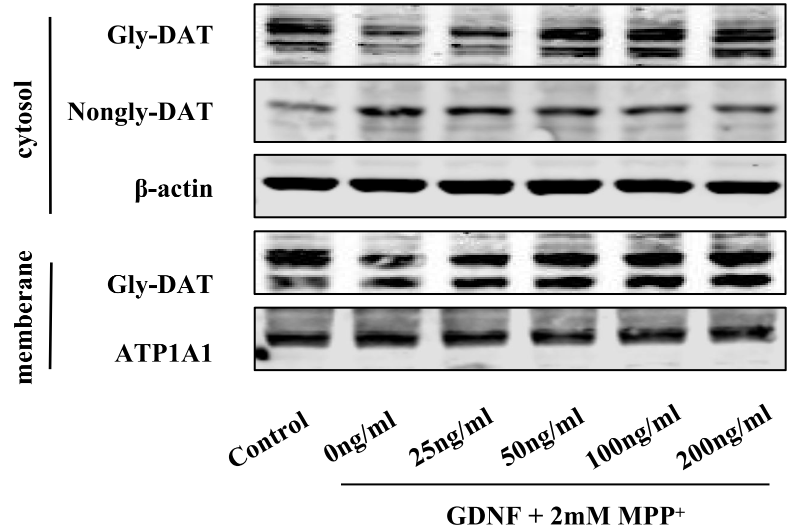
cytosol
Gly-DAT
Nongly-DAT
β-actin
memberane
Gly-DAT
ATP1A1
Control
0ng/ml
25ng/ml
50ng/ml
100ng/ml
200ng/ml
GDNF + 2mM MPP+

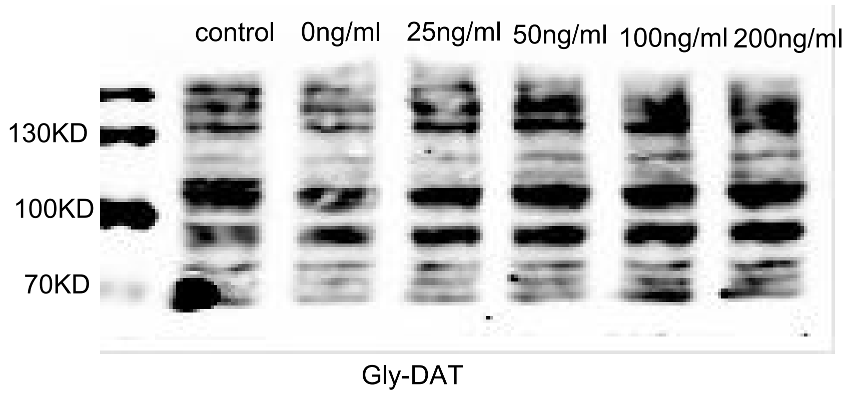
control
0ng/ml
25ng/ml
50ng/ml
100ng/ml
200ng/ml
130KD
100KD
70KD
Gly-DAT

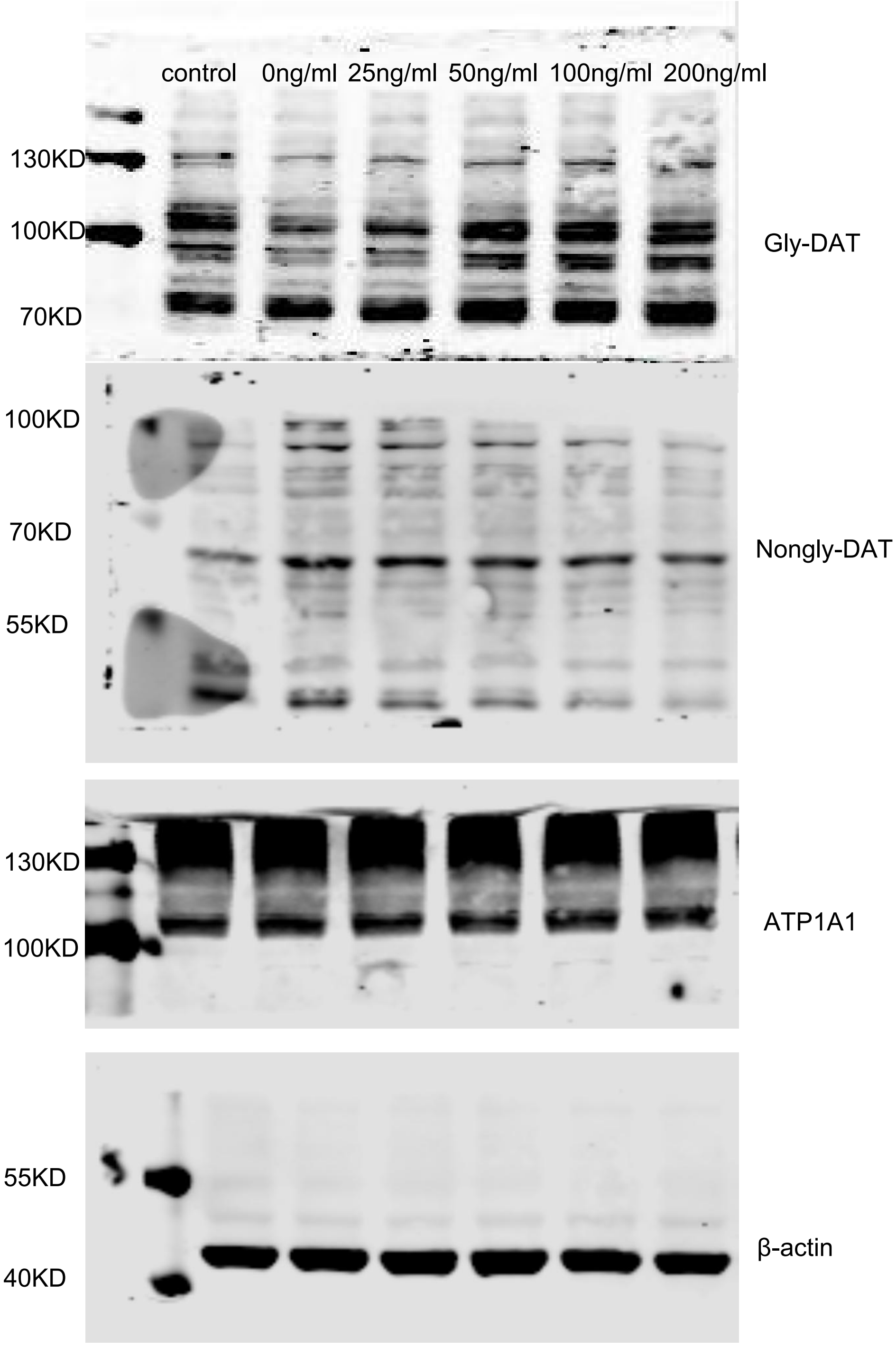
control
0ng/ml
25ng/ml
50ng/ml
100ng/ml
200ng/ml
130KD
100KD
70KD
Gly-DAT
100KD
70KD
55KD
Nongly-DAT
130KD
100KD
ATP1A1
55KD
40KD
β-actin

fig1: C

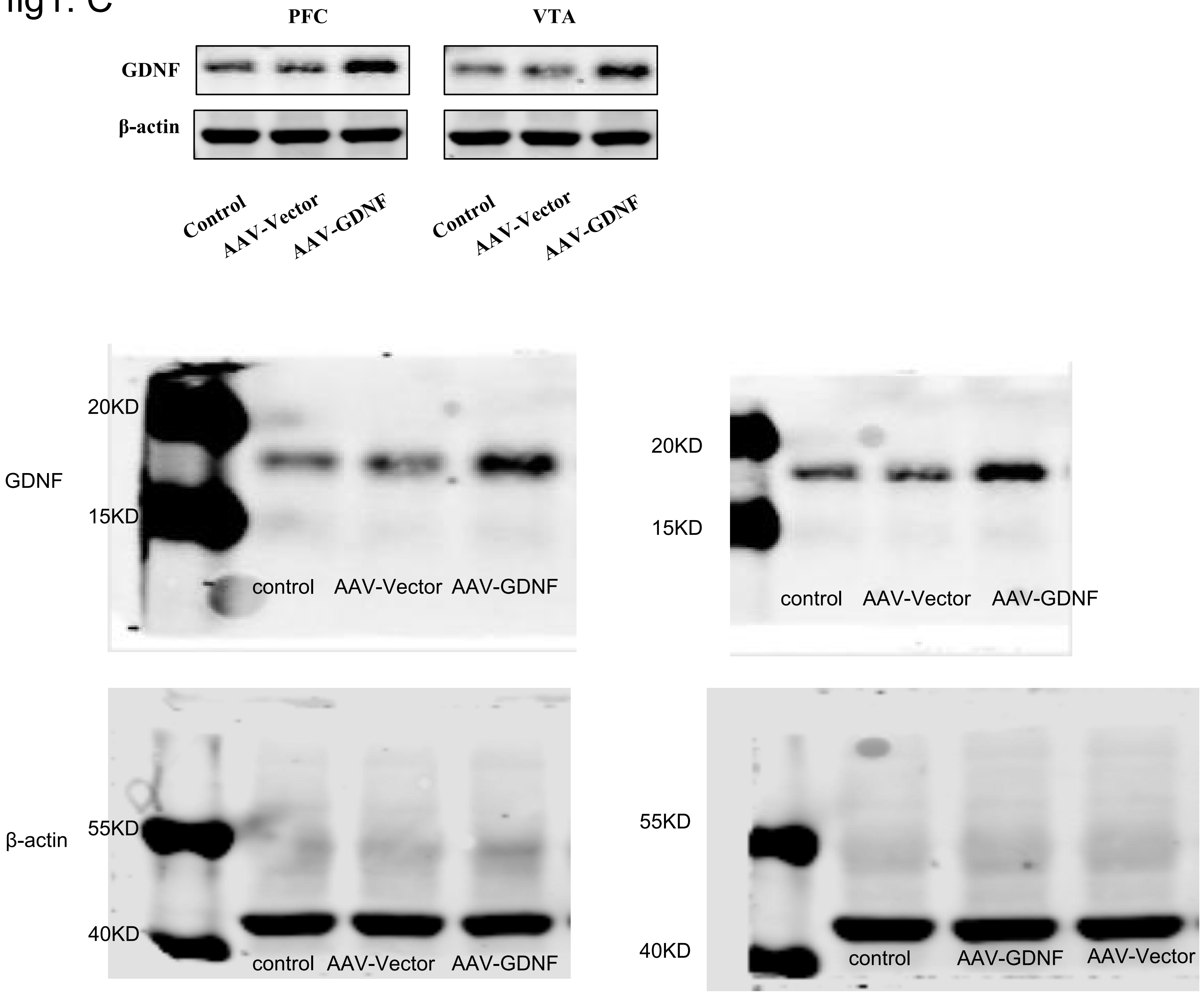
PFC
VTA
GDNF
β-actin
Control
AAV-Vector
AAV-GDNF
Control
AAV-Vector
AAV-GDNF
20KD
15KD
GDNF
control
AAV-Vector
AAV-GDNF
20KD
15KD
control
AAV-Vector
AAV-GDNF
β-actin
55KD
40KD
control
AAV-Vector
AAV-GDNF
55KD
40KD
control
AAV-GDNF
AAV-Vector

fig1: D (PFC)

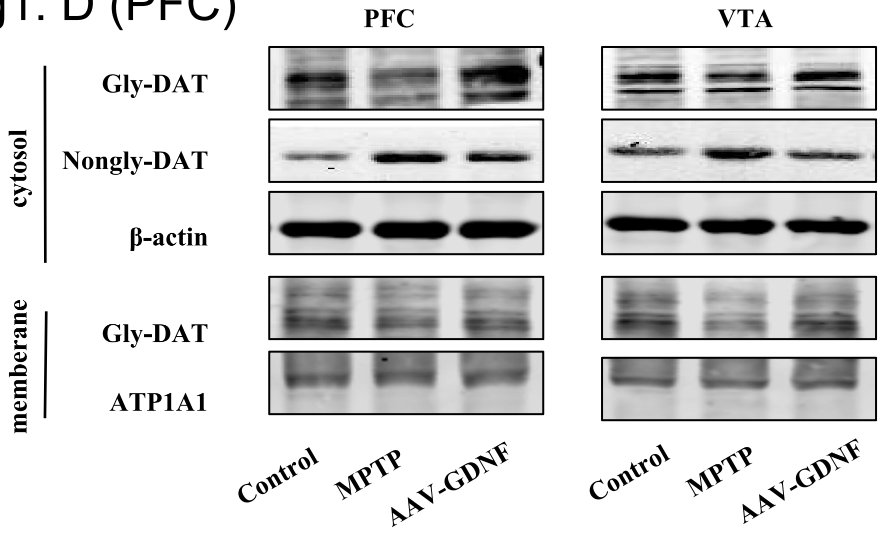

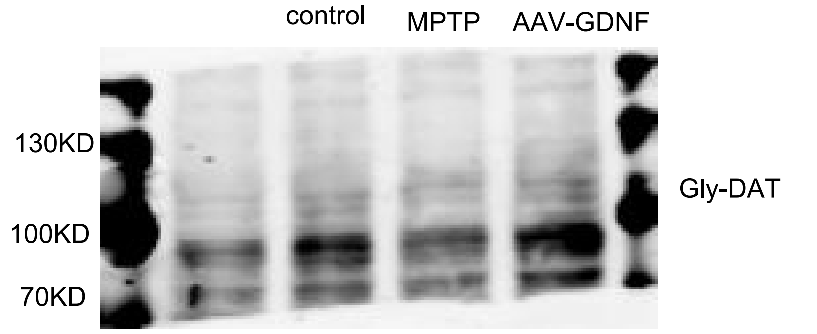

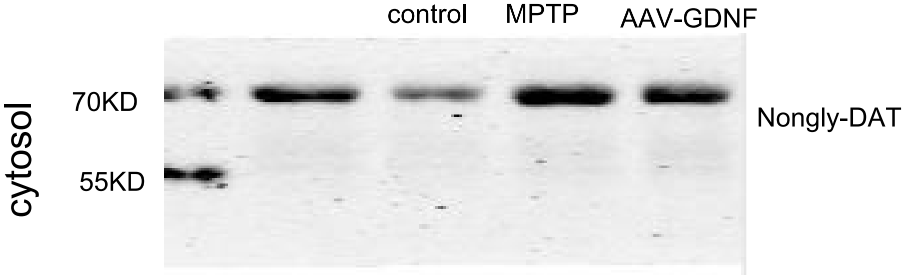

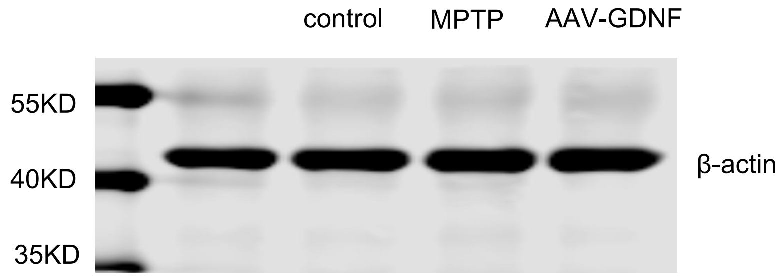

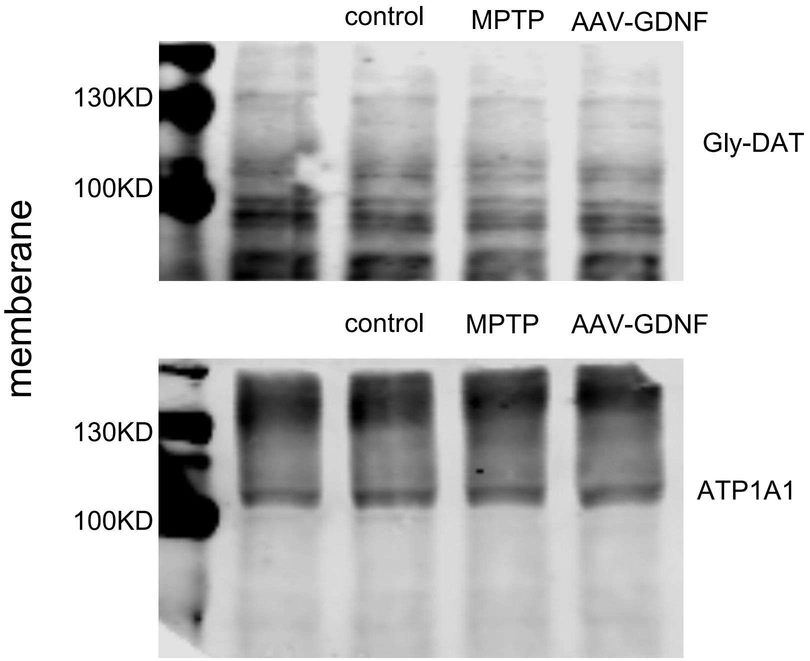

Remark: The first column is waste sample group, the results are not for reference, the purpose is to flatten the strip.

fig1: D (VTA)

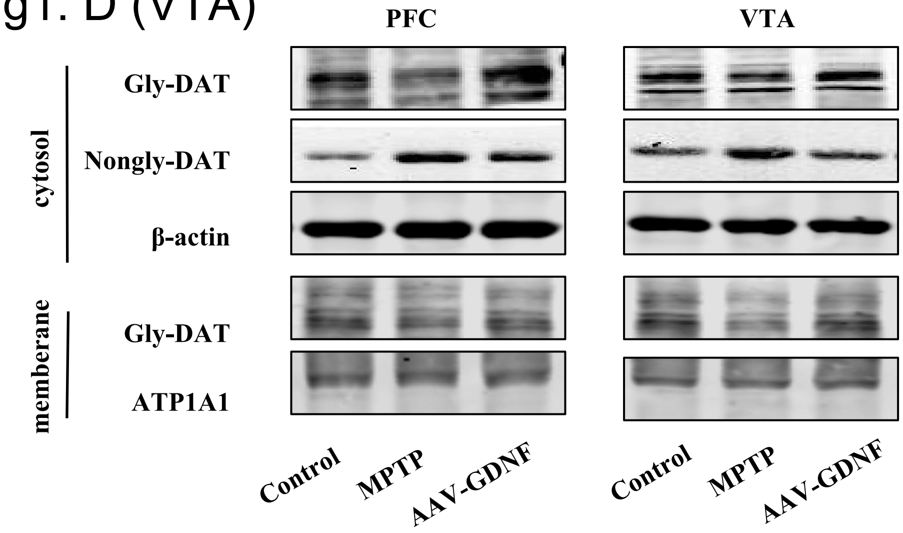

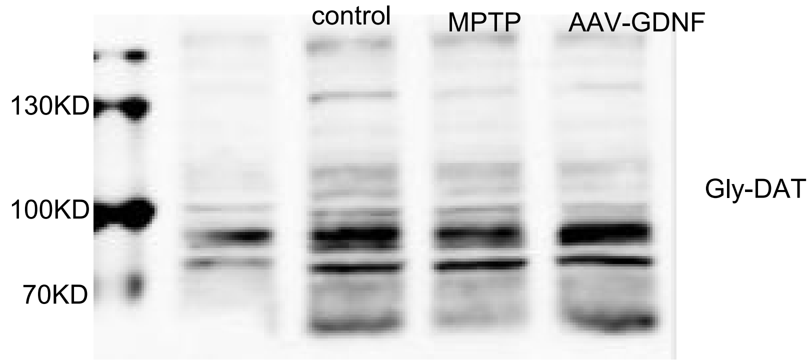

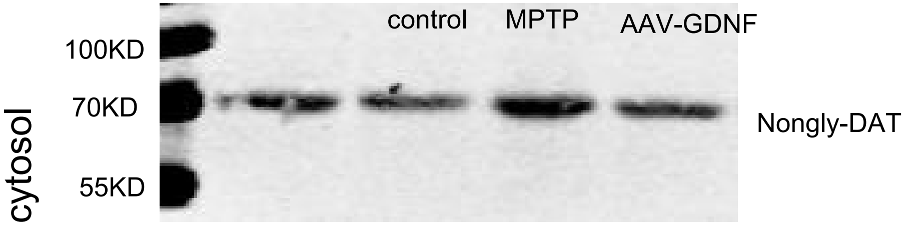

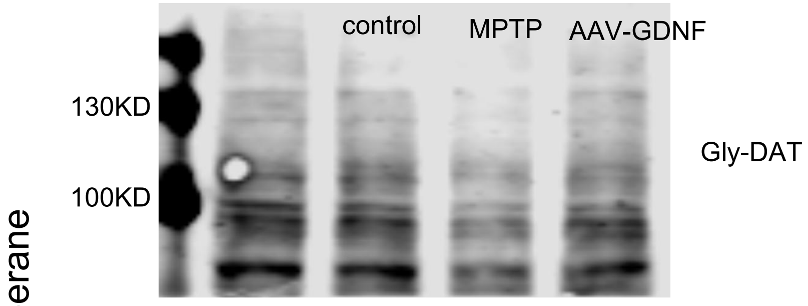

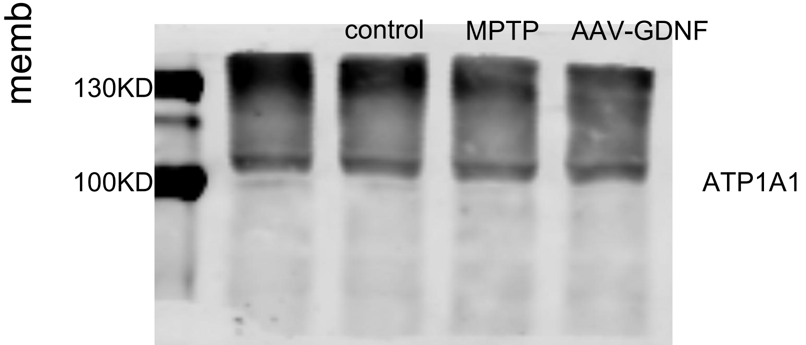

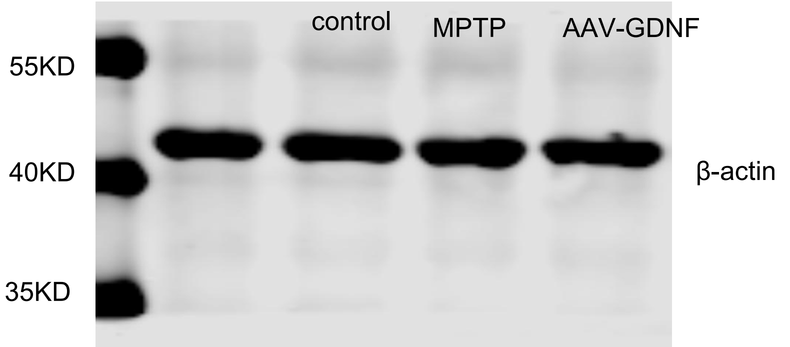

Remark: The first column is waste sample group, the results are not for reference, the purpose is to flatten the strip.

fig 2: B

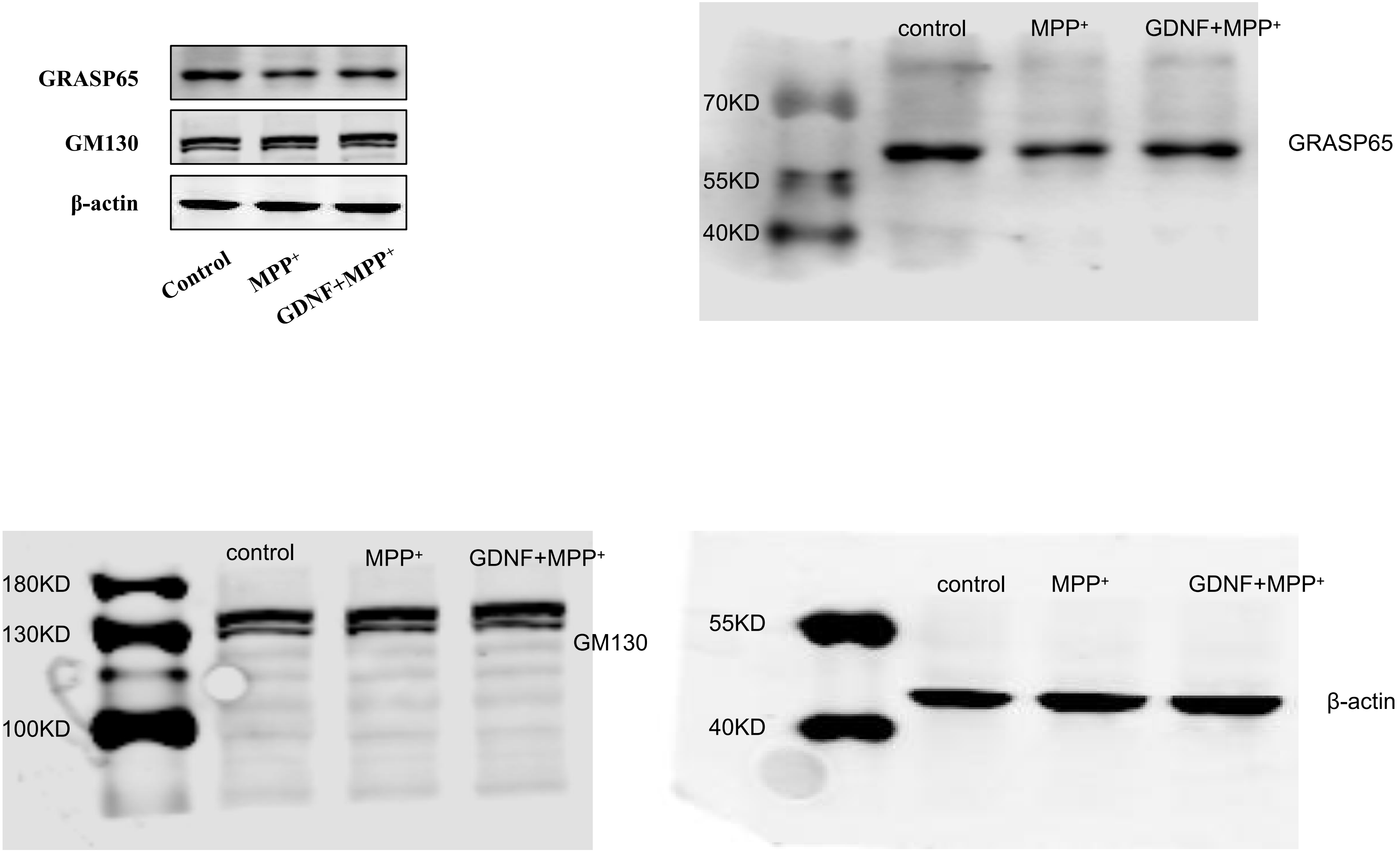
GRASP65
GM130
β-actin
Control
MPP+
GDNF+MPP+
control
MPP+
GDNF+MPP+
70KD
55KD
40KD
GRASP65
control
MPP+
GDNF+MPP+
180KD
130KD
100KD
GM130
control
MPP+
GDNF+MPP+
55KD
40KD
β-actin

fig 2: E

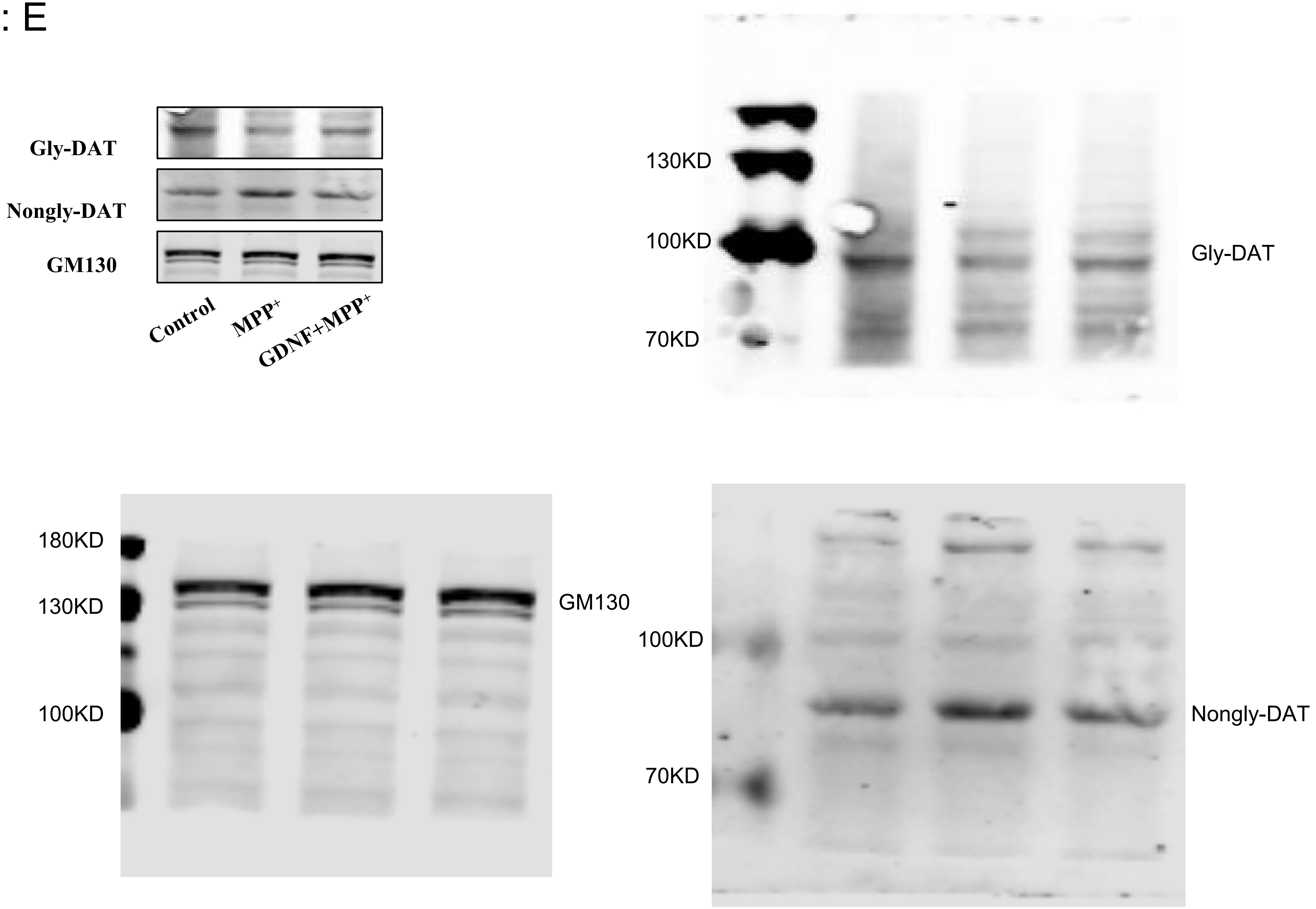

Gly-DAT
Nongly-DAT
GM130
Control
MPP+
GDNF+MPP+
130KD
100KD
70KD
Gly-DAT
180KD
130KD
100KD
GM130
100KD
70KD
Nongly-DAT

fig 2: F (PFC)

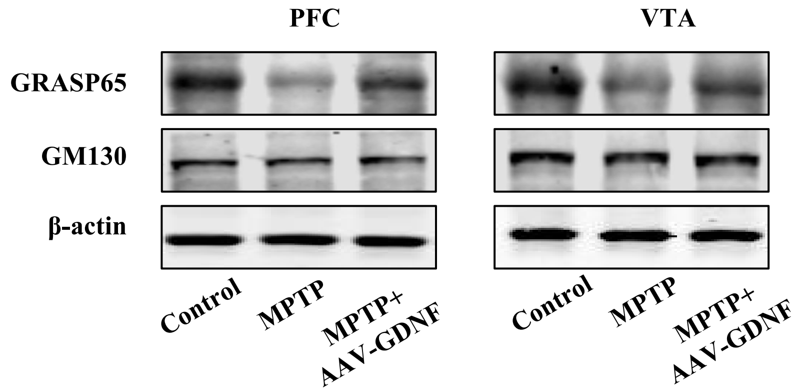

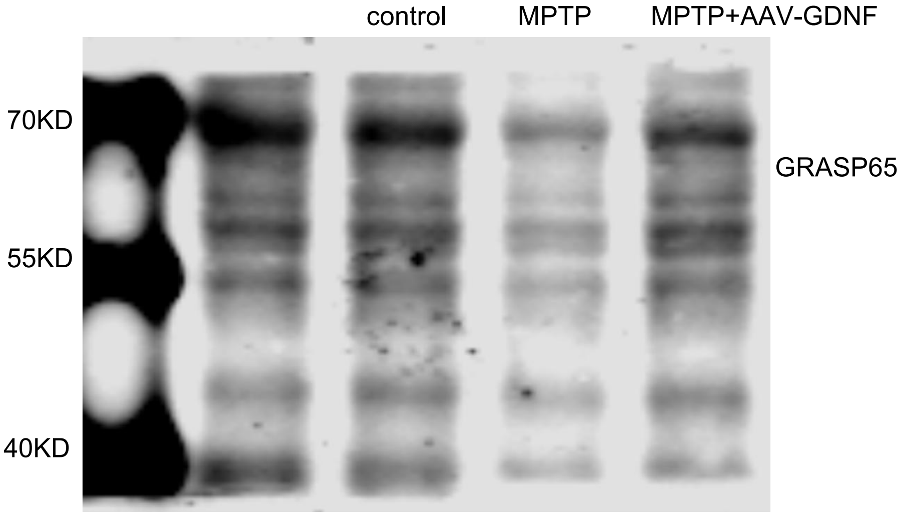

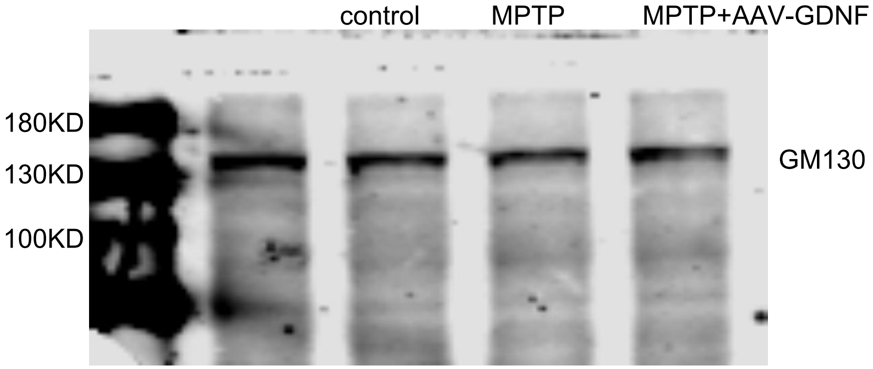

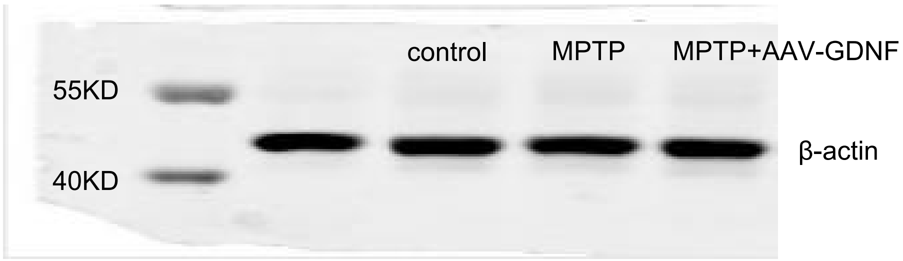

Remark: The first column is waste sample group, the results are not for reference, the purpose is to flatten the strip.

fig 2: F (VTA)

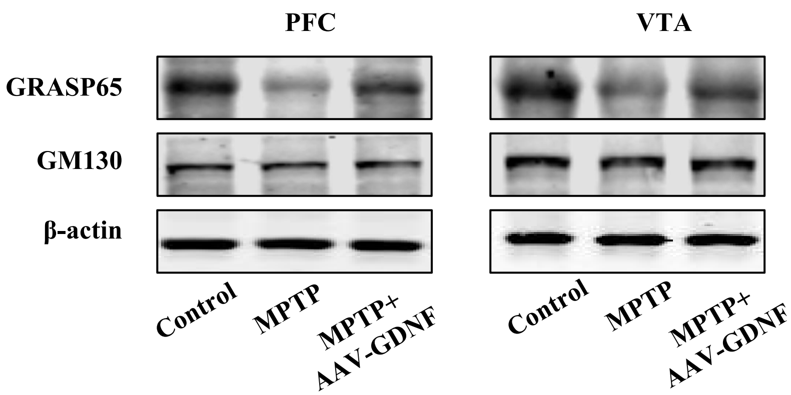

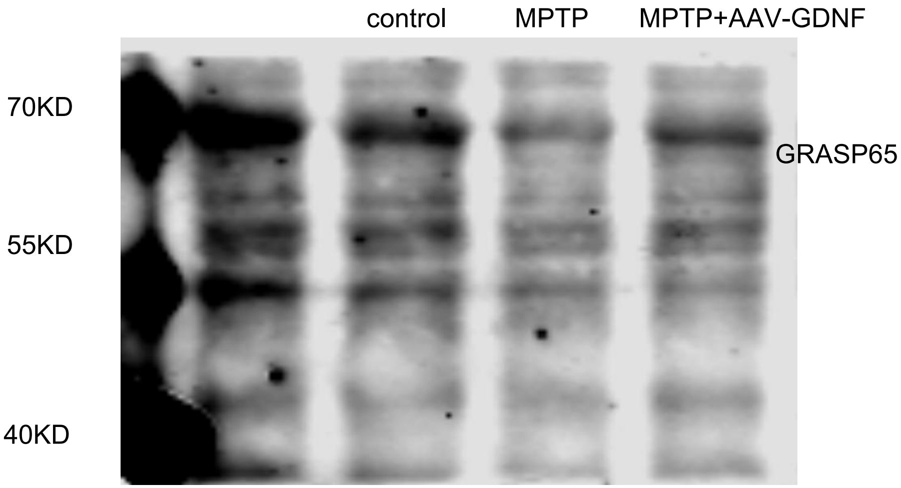

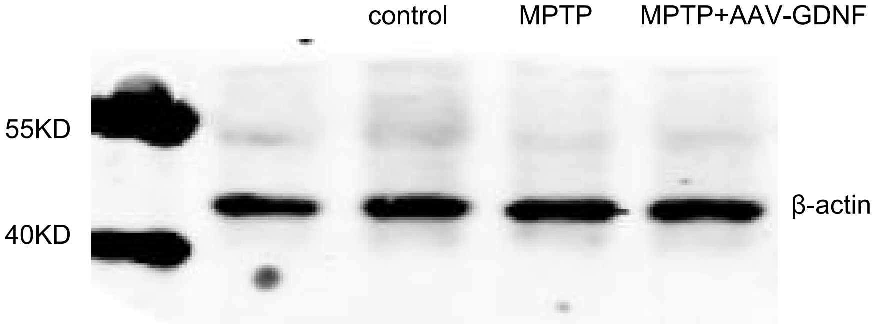

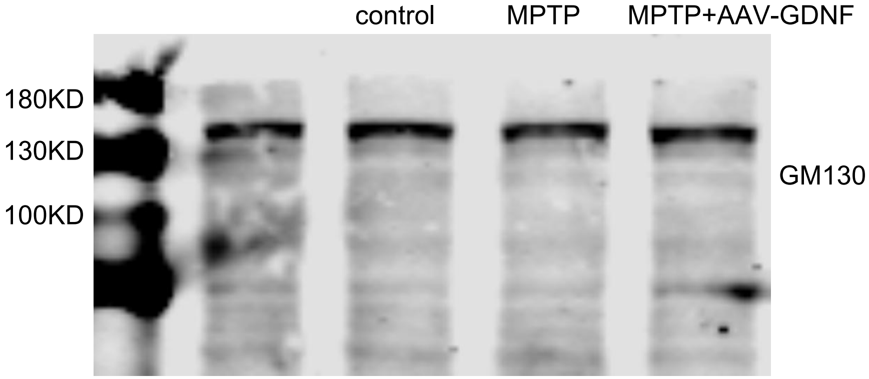

Remark: The first column is waste sample group, the results are not for reference, the purpose is to flatten the strip.

fig 3: A

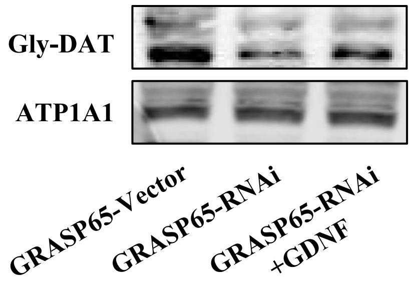
Gly-DAT
ATP1A1
GRASP65-Vector
GRASP65-RNAi
GRASP65-RNAi
+GDNF

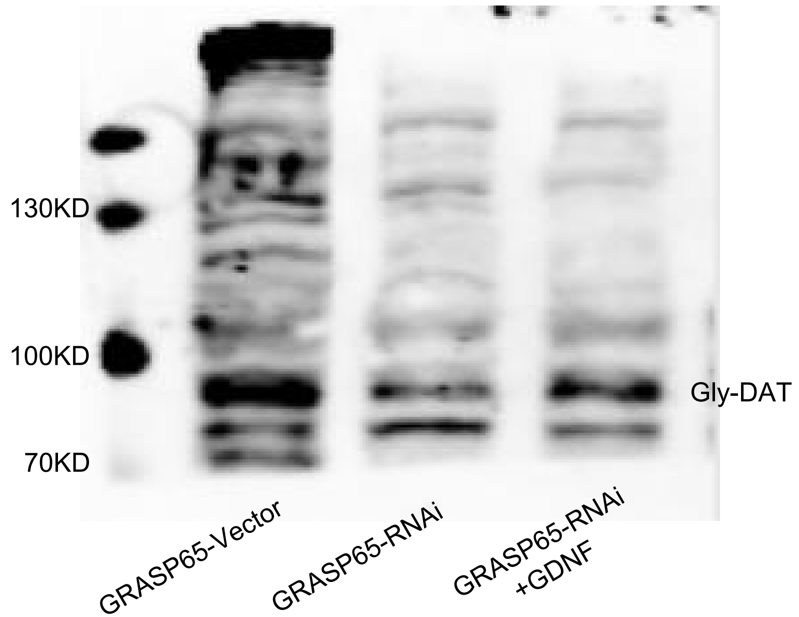
130KD
100KD
70KD
Gly-DAT
GRASP65-Vector
GRASP65-RNAi
GRASP65-RNAi
+GDNF

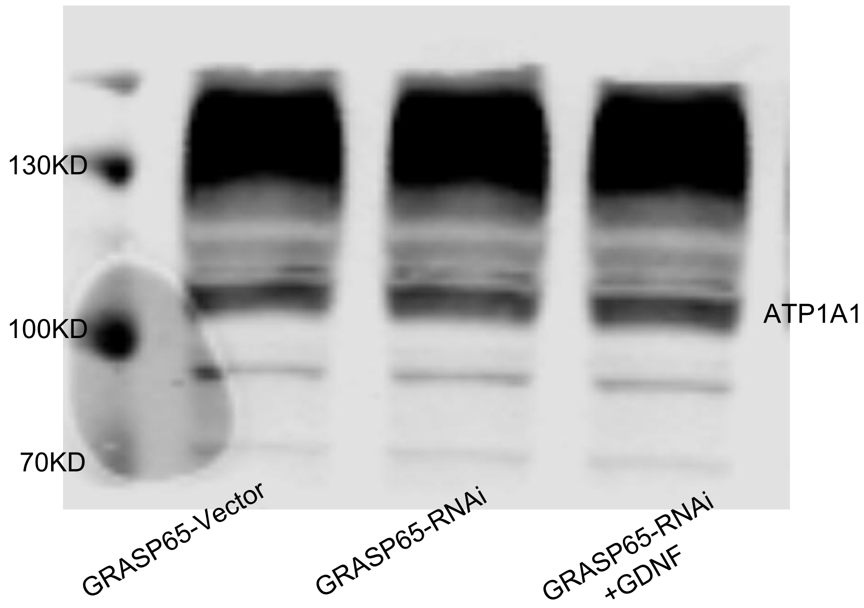
130KD
100KD
70KD
ATP1A1
GRASP65-Vector
GRASP65-RNAi
GRASP65-RNAi
+GDNF

fig 3: B

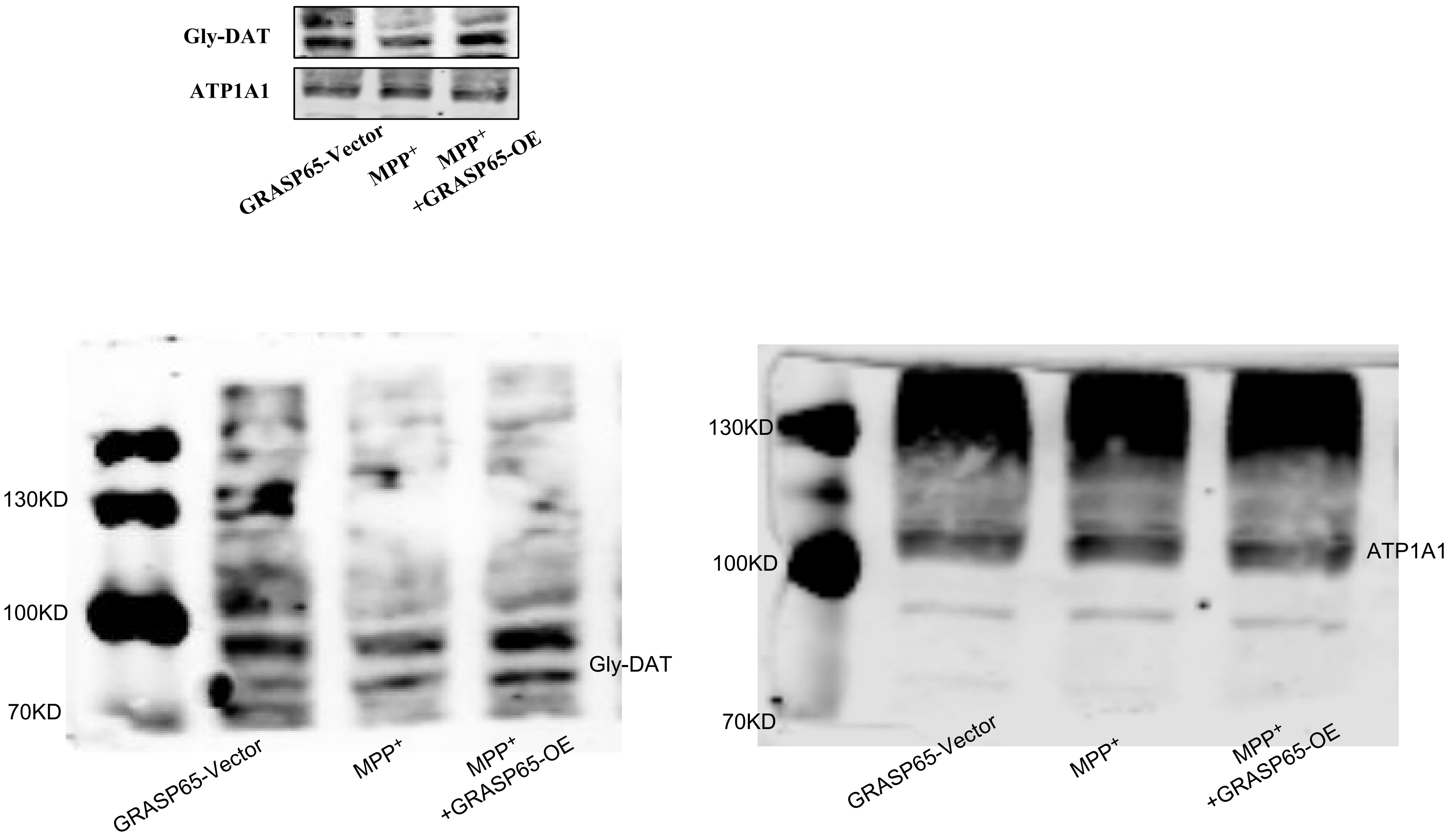
Gly-DAT
ATP1A1
GRASP65-Vector
MPP+
MPP+
+GRASP65-OE
130KD
100KD
70KD
Gly-DAT
GRASP65-Vector
MPP+
MPP+
+GRASP65-OE
130KD
100KD
70KD
ATP1A1
GRASP65-Vector
MPP+
MPP+
+GRASP65-OE

Supplementary Figure1: B

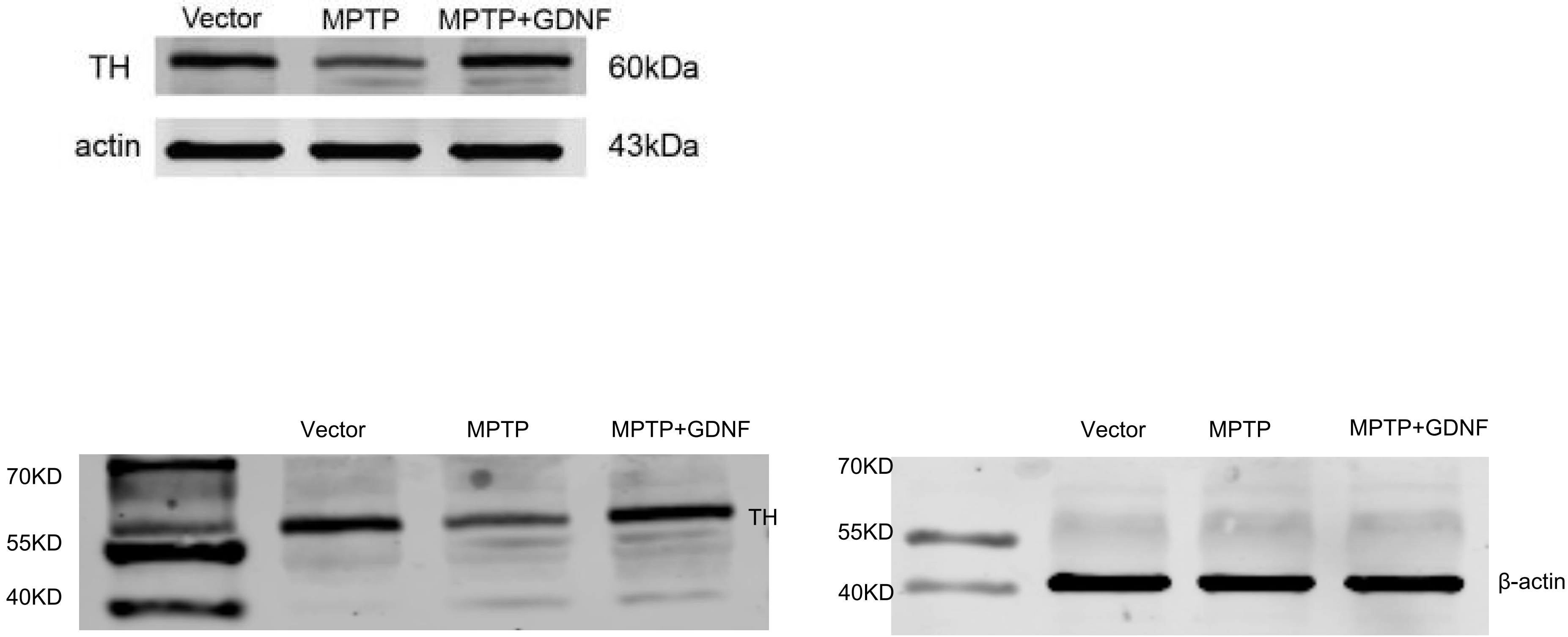

Supplement: Supplementary file 2 — Supplementary Figures. [file 41598_2024_68609_MOESM2_ESM.pdf]
