# Supplementary figures and images for: GDNF improves the cognitive ability of PD mice by promoting glycosylation and membrane distribution of DAT
Source: Sci Rep. 2024 Aug 1;14:17845. doi: 10.1038/s41598-024-68609-y (PMC11294596; doi:10.1038/s41598-024-68609-y)

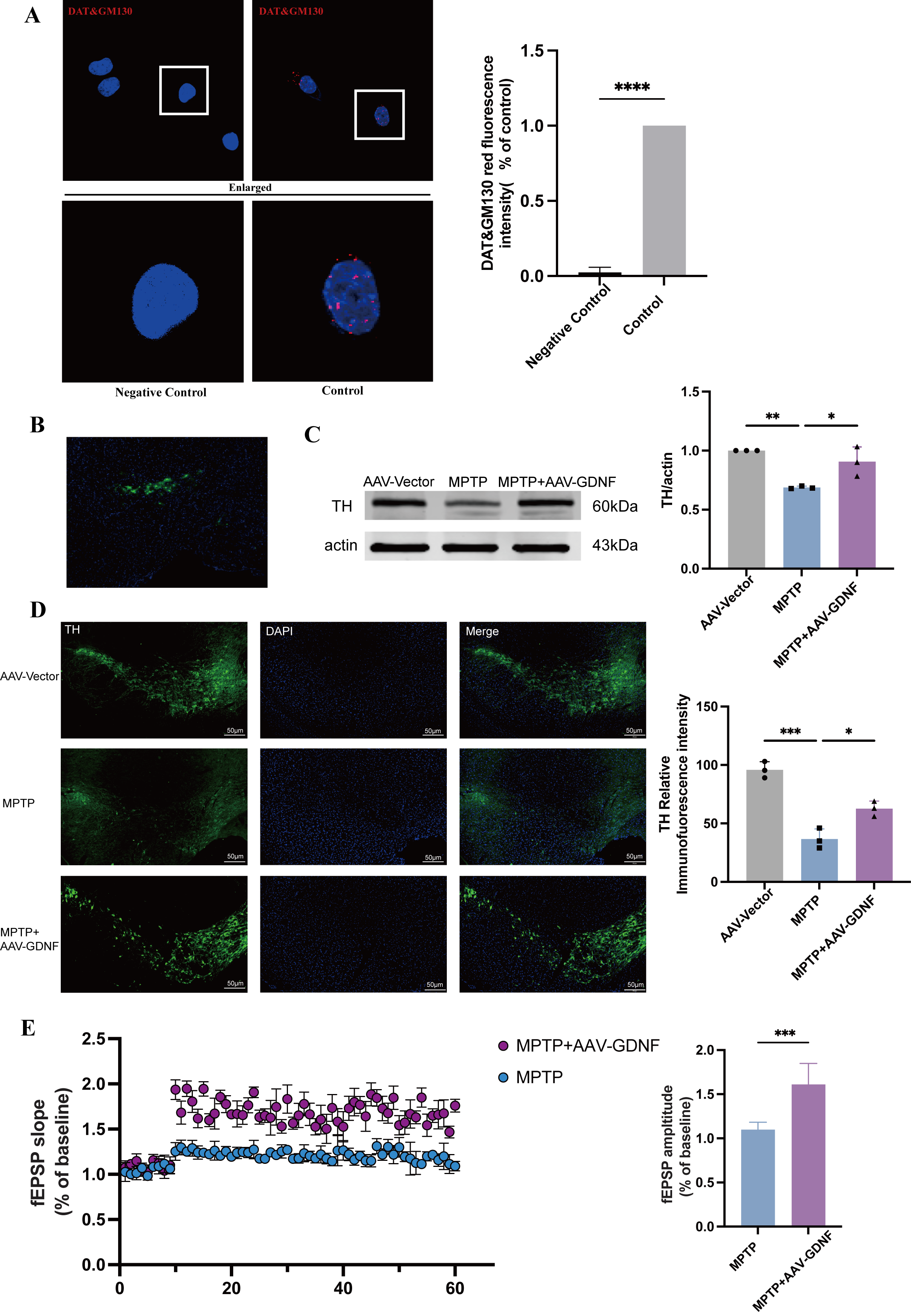

Supplement: Supplementary file 3 — Supplementary Figure 1. [file 41598_2024_68609_MOESM3_ESM.tif]
